# Development and testing of an instrument to measure contextual factors influencing self-care decisions among adults with chronic illness

**DOI:** 10.1186/s12955-022-01990-2

**Published:** 2022-05-23

**Authors:** Shayleigh Dickson Page, Christopher Lee, Subhash Aryal, Kenneth Freedland, Anna Stromberg, Ercole Vellone, Heleen Westland, Douglas J. Wiebe, Tiny Jaarsma, Barbara Riegel

**Affiliations:** 1 University of Pennsylvania School of Nursing, Philadelphia, PA US; 2 Boston College William F. Connell School of Nursing, Chestnut Hill, MA US; 3 Mary MacKillop Institute for Health Research, Australian Catholic University, Melbourne, Australia; 4 Washington University School of Medicine, St. Louis, MO US; 5 Department of Health, Medicine and Caring Sciences, Linkoping University, Linkoping, Sweden; 6 University of Rome Tor Vergata, Rome, Italy; 7 University Medical Center Utrecht, Utrecht, the Netherlands; 8 University of Pennsylvania Perelman School of Medicine, Philadelphia, PA US

**Keywords:** Self-care, Decision making, Chronic illness, Instrument development, Psychometrics

## Abstract

**Background:**

Decisions about how to manage bothersome symptoms of chronic illness are complex and influenced by factors related to the patient, their illness, and their environment. Naturalistic decision-making describes decision-making when conditions are dynamically evolving, and the decision maker may be uncertain because the situation is ambiguous and missing information. Contextual factors, including time stress, the perception of high stakes, and input from others may facilitate or complicate decisions about the self-care of symptoms. There is no valid instrument to measure these contextual factors. The purpose of this study was to develop and test a self-report instrument measuring the contextual factors that influence self-care decisions about symptoms.

**Methods:**

Items were drafted from the literature and refined with patient input. Content validity of the instrument was evaluated using a Delphi survey of expert clinicians and researchers, and cognitive interviews with adults with chronic illness. Psychometric testing included exploratory factor analysis to test dimensionality, item response theory-based approaches for item recalibration, confirmatory factor analysis to generate factor determinacy scores, and evaluation of construct validity.

**Results:**

Ten contextual factors influencing decision-making were identified and multiple items per factor were generated. Items were refined based on cognitive interviews with five adults with chronic illness. After a two round Delphi survey of expert clinicians (n = 12) all items had a content validity index of > 0.78. Five additional adults with chronic illness endorsed the relevance, comprehensiveness, and comprehensibility of the inventory during cognitive interviews. Initial psychometric testing (n = 431) revealed a 6-factor multidimensional structure that was further refined for precision, and high multidimensional reliability (0.864). In construct validity testing, there were modest associations with some scales of the Melbourne Decision Making Questionnaire and the Self-Care of Chronic Illness Inventory.

**Conclusion:**

The Self-Care Decisions Inventory is a 27-item self-report instrument that measures the extent to which contextual factors influence decisions about symptoms of chronic illness. The six scales (external, urgency, uncertainty, cognitive/affective, waiting/cue competition, and concealment) reflect naturalistic decision making, have excellent content validity, and demonstrate high multidimensional reliability. Additional testing of the instrument is needed to evaluate clinical utility.

**Supplementary Information:**

The online version contains supplementary material available at 10.1186/s12955-022-01990-2.

## Background

Adults with chronic illness often experience symptoms that interfere with daily life. For example, shortness of breath may limit the distance someone with asthma can walk without taking a break. Self-care of chronic illness includes evaluating changes in physical and emotional signs and symptoms, determining if action is needed, and deciding which action to take [1]. Self-care management involves the implementation and evaluation of the effectiveness of the chosen action (e.g., use inhaler for shortness of breath).

How adults with chronic illness make decisions about what to do when experiencing symptoms is poorly understood. The naturalistic decision making framework may help to explain how such decisions are made. Naturalistic decision making focuses on how people use experience to make decisions and how contextual factors influence this process [2]. The decision maker may experience uncertainty when the situation is ambiguous, the environment is changing, or necessary information is missing. For example, a symptom may be new, or an individual may be unsure what caused the symptom. Decisions may also be influenced by time stress (e.g., symptom changes quickly), the perception that there is much at stake (e.g., symptom is severe), and conflicting input from multiple individuals [2].

Previous work suggests that self-care decisions made by adults fit within the naturalistic decision making framework. In a qualitative analysis, Riegel, Dickson [3] found that the decisions made by adults with chronic heart failure were influenced by experience, decision characteristics (e.g., uncertainty, ambiguity, high stakes, urgency, illness characteristics, and involvement of others in the decision making process), and personal goals. Further, situation awareness (i.e., recognition and interpretation of the symptom) and mental simulation (i.e., mentally thinking through options for “what to do”) were integral to the decision-making process.

In spite of evidence that patients engage in naturalistic decision-making in response to symptoms and that contextual factors influence self-care decisions, there are no valid instruments to measure these factors*.* Instruments are available to assess decision-making style (e.g., spontaneous, intuitive, rational) [4–6] or management of the decision-making process (e.g., coping with decisional conflict) [7, 8]. These instruments are helpful for understanding the patient’s decision-making in general, but they do not assess how contextual factors affect the decision-making process nor are they specific to self-care decisions about symptoms. Measurement of contextual factors influencing self-care decisions about symptoms is important for advancing research in self-care and improving the clinical care of adults with chronic illness*.* If investigators can identify factors that influence self-care decisions, they can design tailored interventions to address specific barriers. The aims of this study were to (i) Develop a theoretically based and clinically relevant self-report instrument that measures contextual factors influencing self-care decisions about symptoms, and (ii) Test its psychometric properties, including dimensionality, construct validity, precision, and reliability.

## Methods

**Fig. 1:**
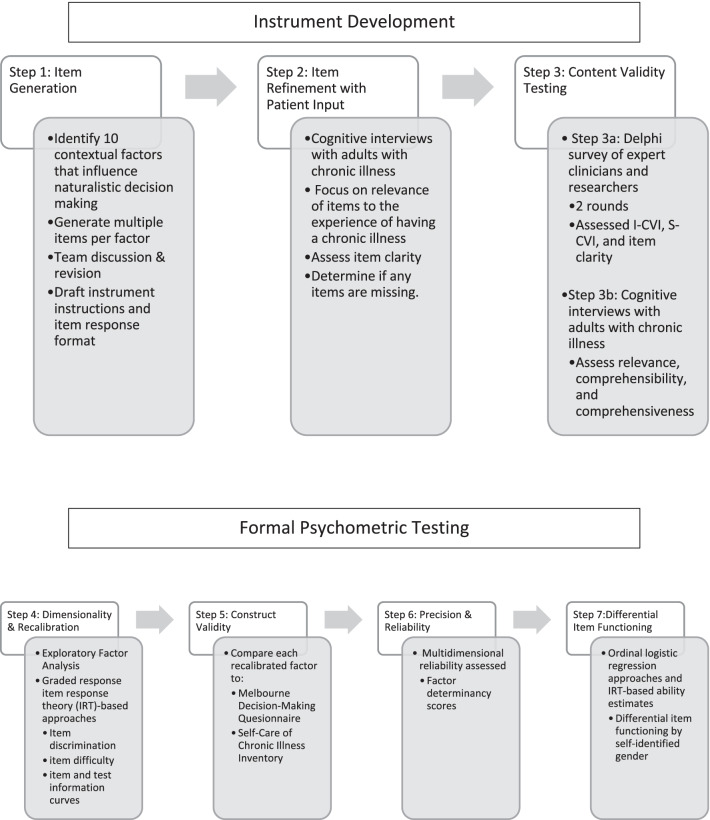
Instrument development and formal psychometric testing process

This study was conducted in two phases: (i) Instrument development and (ii) Formal psychometric testing (Fig. 1).

The COnsensus-based Standards for the selection of health Measurement INstruments (COSMIN; www.cosmin.nl) guided the instrument development and content validity testing, and item response theory guided the initial psychometric testing. Institutional Review Board approval for this study was obtained from the University of Pennsylvania.

## Instrument development

*Step 1: item generation*

First, contextual factors were identified from the literature that are thought to influence self-care decisions. Next, a preliminary list of items was generated. The items described how these contextual factors influence the response to bothersome symptoms based on the foundational work on naturalistic decision making [2] as well as the application of naturalistic decision making to self-care decisions in adults with heart failure [3]. The authors discussed and revised the items as well as the instrument instructions and scoring format until consensus was reached on an initial instrument draft.

*Step 2: item refinement with patient input*

We then conducted cognitive interviews with adults with chronic illness. The purpose of these interviews was three-fold: (1) To assess the relevance of the proposed items to the experience of having a chronic illness, (2) To ensure that patients understood the items, and (3) To improve the comprehensiveness of the instrument by asking if any items were missing. Adults with at least one of five chronic illnesses (arthritis, asthma, chronic obstructive pulmonary disease, diabetes mellitus, and/or heart failure) were recruited through Researchmatch.org, a website supported by the National Institutes of Health on which people from the United States can volunteer to participate in research. These conditions were selected because they are common and often symptomatic. Eligibility criteria included age > 18 years and currently experiencing at least one symptom of a chronic illness. There were no exclusion criteria*.* Interviews were completed by the first author either by phone or video conference. SP, BR, TJ, AS, HW, and EV discussed the results of the cognitive interviews and reached consensus on changes to items.

*Step 3: content validity testing*

Content validity is the degree to which the content of the instrument reflects the construct (i.e., naturalistic decision making) that the instrument was designed to measure [9]. The COSMIN methodology for evaluating content validity defines three properties of content validity (relevance, comprehensiveness, and comprehensibility) and further recommends that both patients and professionals are involved in the validation process [10]. Thus, we evaluated the content validity of the instrument in two ways: (i) A Delphi survey of clinicians and researchers and (ii) Cognitive interviews with adults with chronic illness.

*Step 3a: Delphi survey*

The Delphi technique uses structured questionnaires that are distributed in iterative rounds to a group of experts who remain anonymous to each other throughout the process [11]. For the Delphi survey, we defined experts as (i) Clinicians who routinely help adults make decisions about their chronic illnesses and (ii) Researchers who have published on decision making related to chronic illness in the scientific literature. Experts were identified through a Facebook discussion on the topic of decision-making in self-care, a literature search on decision-making in chronic illness, and the professional networks of the study authors. The Delphi survey was completed electronically using Qualtrics (Provo, UT). Respondents rated the *relevance* of items to the construct of naturalistic decision making on a 4-point scale (not relevant, somewhat relevant, quite relevant, highly relevant). The *comprehensibility* of items was rated dichotomously (clear, not clear). Respondents had the opportunity to suggest new items to support *comprehensiveness* of the instrument and ensure that no facets of the construct were omitted. Finally, respondents provided feedback on the clarity of the proposed instrument instructions and the scoring format.

After each round, the Content Validity Index (CVI) of each item (I-CVI) was calculated by dividing the number of respondents reporting that an item was “quite relevant” or “highly relevant” by the total number of respondents [12]. An I-CVI greater than 0.78 is considered evidence of good content validity [12]. Thus, to be retained without revision, the I-CVI had to be 0.78 or higher. Consensus on clarity was defined as at least 75% of the respondents agreeing that the item was clear. SP, BR, TJ, AS, HW, and EV met to discuss responses following each round of the Delphi survey. Items were retained, revised, or deleted following discussion of the I-CVI and clarity data as well as the respondents’ open-ended suggestions.

The Content Validity of the Scale (S-CVI) was calculated at the conclusion of the Delphi survey. We report the average of the I-CVIs for all items on the scale (i.e., S-CVI/Ave). According to Polit, Beck [12], a S-CVI/Ave greater than 0.90 indicates excellent content validity.

*Step 3b: cognitive interviews*

Following the Delphi survey, cognitive interviews with a second set of adults with chronic illness were completed to ensure that the revised items remained relevant to their experience and to assess comprehensiveness and comprehensibility of the instrument. Participants were again recruited through Researchmatch.org using the same inclusion criteria previously described. Participants were read the instrument instructions followed by each item. Per the instrument instruction, they rated how much the item influenced their decision on a 5-point Likert Scale from “not at all” to “a great deal”. Participants were encouraged to “think aloud” and describe how they arrived at each answer. They also provided feedback on the clarity of the instrument instructions and Likert scale. To elicit more information, three types of verbal probing techniques were used: 1) comprehensiveness/ interpretation probes (e.g., why do you think…?), 2) paraphrasing (e.g., please repeat that statement in your own words), 3) general probes (e.g., how did you arrive at that answer?) [13].

## Formal psychometric testing

### Sample

Participants were recruited through Reaserchmatch.org for psychometric testing of the newly developed **Self- Care Decisions Inventory**. Invitations to participate were sent to adults (age > 18y) with at least one chronic condition. Chronic condition was defined as any of the symptomatic physical or mental health conditions that are included on the list of chronic conditions published by the Office of the Assistant Secretary for Health in the Department of Health and Human Services of the United States [14]. Additional eligibility criteria included currently experiencing at least one symptom of the chronic illness. Surveys were completed electronically using Qualtrics (Provo, UT).

*Step 4: dimensionality & recalibration*

Descriptive statistics of central tendency and dispersion were used to describe the sample. Exploratory factor analysis was used to test dimensionality; response options were handled as ordered categorical data, and weighted least squares mean and variance adjustment and geomin oblique rotation (with a primary loading cutoff > 0.40, and significant loading (p < 0.05) on alternative factors) were used [15]. Models ranging from 1 to 8 factors were compared using cutoff values of model fit (i.e., root mean square error of approximation (RMSEA) < 0.05, comparative fit index (CFI) and Tucker-Lewis index (TLI) of ≥ 0.95, and standardized root mean square residual (SRMR) < 0.08) [16, 17]. Velicer’s minimum average partial correlation was calculated post-estimation along with Horn’s parallel analysis to confirm the number of factors [18–20], assuming that a correctly identified multidimensional model also can result in local independence [21].

Graded response item response theory (IRT)-based approaches were used within each factor for recalibration using information on a) item discrimination (slope and significance), b) item difficulty (graded response model slopes and standard errors as well as boundary and category characteristic curves), as well as c) item and test information (item and test information curves) [22].

*Step 5: construct validity*

No measure of the contextual factors influencing decision making as described in the naturalistic decision making framework exists, so we chose to assess convergent validity, the degree to which the new measure is related to other measures of decision-making. We compared each recalibrated Self-Care Decisions Inventory with the Melbourne Decision-Making questionnaire (Melbourne DMQ) domains. The Melbourne DMQ measures four patterns for coping with decisional conflict: vigilance, hypervigilance, buck passing and procrastination [7]. The coping pattern of vigilance involves clarifying objectives, canvassing an array of alternatives, searching for relevant information, assimilating that information, and evaluating alternatives before making a choice. The pattern of hypervigilance involves frantic searching, time pressure, and impulsive choice of a contrived solution. Buck passing is described as an avoidance style associated with defensiveness and dependency. Finally, procrastination is another form of defensive avoidance that involves delaying decision making. Higher scores indicate a preference for that coping pattern and vigilance is negatively correlated with the other patterns. The scale alpha coefficient reliabilities ranged from 0.74 to 0.87 in a sample of 2018 participants from six countries [7]. We hypothesized that each recalibrated scale on the Self-Care Decisions Inventory would be significantly associated with Melbourne DMQ domains. Linear correlations with Bonferroni correction were computed to test these hypotheses.

Criterion validity is the extent to which one measure predicts scores on another measure. To evaluate criterion validity, we assessed the degree to which scores on the Self-Care Decisions Inventory predict adequate self-care, using the Self-Care of Chronic Illness Inventory (SC-CII), a 20-item self-report generic measure of self-care based on the Theory of Self-Care of Chronic Illness [23]. The SC-CII includes three scales: Self-Care Maintenance, Self-Care Monitoring, and Self-Care Management. Scores range from 0 to 100 and higher scores indicate better self-care. A cut-point of ≥ 70 is used to indicate self-care adequacy on each scale [24]. The Self-Care Management scale is multidimensional, thus reliability is calculated using the global reliability index [25]. Reliability of this scale was 0.71 in a sample of 400 adults with chronic illness [23]. We hypothesized that adequate self-care management would be positively associated with the ‘Urgency’ scale in the Self-Care Decisions Inventory and negatively associated with the Self-Care Decisions Inventory ‘Uncertainty’ scale, discussed further below. Scores on the Self-Care Decisions Inventory were standardized to range from 0–100. Two-sample t-tests were used to compare Self-Care Decisions Inventory scores between groups of individuals with adequate and inadequate self-care management. Hedge’s g is reported for effect size.

*Step 6: precision & reliability*

IRT test information function curves were generated to display the range of each construct where recalibrated scales of the Self-Care Decisions Inventory are most accurate. Multidimensional reliability was quantified using factor determinacy scores for the recalibrated Self-Care Decisions Inventory in confirmatory factor analysis.

*Step 7: differential item functioning*

Ordinal logistic regression approaches were combined with IRT-based ability estimates to detect differential item functioning related to self-identified gender [26].

Factor analyses were performed in Mplus v8 (Los Angeles, CA), and IRT models and validity testing were performed in Stata v16 (College Station, TX). Full information maximum likelihood estimation (FIML) was used to impute the 0.3% of data that were missing at random.

## Results

### Instrument development

*Step 1: item generation*

**Fig. 2:**
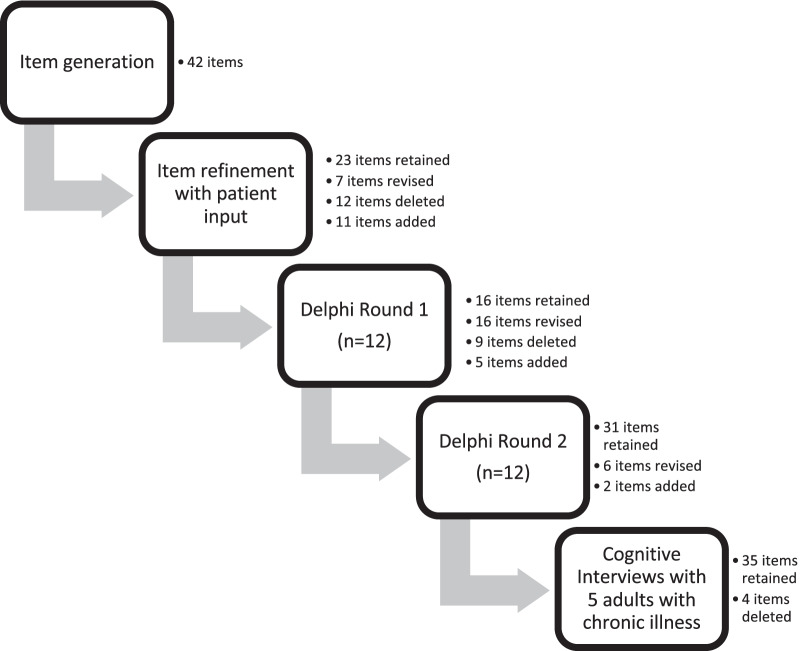
Flow chart of item selection and revision for the Self-Care Decisions Inventory. This flowchart displays the process of item development. Initially 42 items were generated. Items were subsequently retained, revised, added, or deleted based on patient input, a two round Delphi survey, and cognitive interviews with adults with chronic illness

The instrument instructions directed survey respondents to think about the last time that they had a bothersome symptom of their chronic illness and then rate how much each item influenced their decision about what to do in response to that symptom. Ten contextual factors were derived from the literature: prior experience, competing personal goals, uncertainty and ambiguity, urgency, situation awareness, involvement of multiple individuals, interpretation of symptom meaning, illness characteristics, dynamically evolving conditions, and high stakes [2, 3]. Several items were generated for each contextual factor, resulting in an initial draft of 42 items. From August to October 2020, the investigators discussed and revised items. Consensus discussions centered on ensuring that all contextual factors were adequately represented and that items were clearly worded. For example, for prior experience*,* we decided to include items that captured both having experience (e.g., *I thought about similar past decisions*) and lack of experience (e.g., *the symptom was new to me*). Each item was rated on a 5-point Likert scale with response options of not at all (1), a little (2), some (3), a lot (4), and a great deal (5). Figure 2 displays the process of item selection and revision.

*Step 2* *:* *item refinement with patient input*

Five women, ages 43–71, completed the cognitive interviews. Each had multiple chronic conditions and had been living with at least one symptomatic chronic illness for more than 10 years. One participant reported having both physical and mental illnesses.

Based on the responses of these adults, 23 items were retained as initially written, 7 items were revised, and 11 items were added. Item revisions were made to improve clarity. For example, “*I recognized this from last time”* was changed to “*I recognized this symptom from last time”*. Items were added when participants identified that a factor that influences their decision was not captured by existing items. For example, a participant identified that her decision making is affected by depressive symptoms, so the item “*I felt too down, so I put off making a decision”* was added. Finally, 12 items were deleted as irrelevant (8 items) or redundant (4 items). The refined draft of the instrument included 41 items.

*Step 3a: Delphi survey*

Twenty-six experts were invited via email to complete the Delphi survey. There were 12 respondents (9 female, 3 male) in round 1 and all 12 respondents also completed round 2. Experts were from United States (n = 7), Italy (n = 4), and Germany (n = 1). All experts reported that their primary role was as a professor/lecturer at university and ten also reported clinical experience. The average number of years of experience, specifically in the clinical care of adults with chronic conditions was 16 years (range: 4–44). Eleven out of 12 experts had a PhD and one had a master’s degree.

I-CVI and clarity data for each Delphi round are summarized in Table 1. In round 1, I-CVIs ranged from 0.5 to 1.0. Two items, “I didn’t want to look weak” (I-CVI = 0.5) and “I knew I was in trouble” (I-CVI = 0.75) were rated as irrelevant and also had less than 75% agreement on clarity, thus both items were deleted. Twelve items did not reach consensus on clarity (i.e., rated as clear by < 75% experts). Six of these items were deleted because there were other items that evaluated the same contextual factor and scored better in terms of clarity. Six of these items were revised and were subsequently rated as clear by ≥ 75% of experts in the second round. Five items were added and one was deleted based on the open-ended feedback in this round.

**Table 1:** I-CVI and clarity data by Delphi round

	# of experts	# of items in round	I-CVI^a^ range	% of I-CVI ≥ 0.78^b^	Clarity^c^ range	Items rated as clear by < 75% of experts^d^	Items deleted or added in round
Round 1	12	41	0.5 – 1.0	95%	42–100%	12 items	9 deleted 5 added
Round 2	12	37	0.83—1.0	100%	75–100%	0 items	2 added

^a^Item Content Validity Index (I-CVI) = number of respondents who rated the item as ‘highly relevant’ or ‘quite relevant’ divided by total number of respondents

^b^An I-CVI of 0.78 or higher indicates good content validity at the item level

^c^Clarity = number of respondents who rated the item as clear divided by the total number of respondents

^d^Items rated as clear by less than 75% of experts required revision

In round 2, the I-CVIs were 1.0 for 26 items, 0.92 for 9 items, and 0.83 for 2 items. All items were rated as clear by ≥ 75% of experts. Based on the open-ended feedback provided by experts, minor revisions to the wording were made to 6 items and 2 items were added.

The Delphi survey was closed after the second round as consensus on item relevance and clarity was achieved. The S-CVI/Ave of this 39-item instrument was excellent at 0.92.

*Step 3b: cognitive interviews*

Five adults (3 female, 2 male), ages 44–70, completed the second round of cognitive interviews. Four adults had multiple chronic conditions, including one who reported both physical and mental health conditions. Two adults had been diagnosed in the last 3 years, while three adults reported having at least one symptomatic chronic condition for more than 10 years. Despite having chronic conditions for multiple years, one adult was experiencing a new symptom and spoke about decision-making for this new symptom during the cognitive interview.

In these cognitive interviews, respondents reported that items were relevant to their experience and the instrument was comprehensive. No new items were suggested. For three items, participants reported confusion about wording and endorsed multiple interpretations of the item. These three items were deleted because there were other items that captured the same contextual factor and were clearer to participants. One item, “*I worried about the cost of treatment*”, was deleted based on participant feedback. Participants discussed that worries about cost were directly tied to whether they had adequate insurance coverage. Thus, the item reflected access to insurance coverage rather than a factor that influenced decision making. We aimed to develop an instrument that could be used internationally and since insurance coverage and treatment costs differ across countries, we chose to delete this item. The instrument instructions were also shortened and simplified based on participant feedback. The anchors of the 5-point scale were changed to “No Influence” [1] and “A Lot of Influence” [5]. Following content validity testing, the instrument contained 35 items.

### Psychometric testing

Invitations to participate were sent to 1,127 individuals who expressed interested in the study on Researchmatch.org. A total of 431 individuals completed the survey for a response rate of 38.2%. The typical participant was female, White, non-Hispanic, with at least some college education (Table 2). The sample was diverse in terms of the types of chronic conditions, including 22.5% who self-reported having a mental health condition. The Self-Care Decision Inventory instructs participants to think about the last time that they had a worrisome symptom and participants provided a free-text response to the question “what symptom are you thinking about?” Most participants (n = 356, 82.6%) reported a single symptom, while 44 (10.2%) reported multiple symptoms. The most frequently reported symptoms were pain (28.1%), respiratory symptoms (11.5%), mental health symptoms (7.4%), fatigue (7.1%), and gastrointestinal symptoms (7.1%).

**Table 2:** Participant characteristics (n = 431)

	n (%)
Age	
mean (sd)	54.93 (16.15)
Gender (n = 426)	
Female	302 (70.1)
Race (n = 425)	
White	375 (87)
Black	20 (4.6)
Native American/Alaska Native	2 (0.5)
Asian	6 (1.4)
Native Hawaiian/Pacific Islander	1 (0.2)
Mixed (two or more)	21 (4.9)
Ethnicity (n = 427)	
Hispanic	19 (4.4)
Education (n = 430)	
High school or less	23 (5.3)
Some college	74 (17.2)
Associate’s or Bachelor’s degree	179 (41.5)
Master’s degree	102 (23.7)
Professional or doctoral degree	45 (10.4)
Other	7 (1.6)
Employment (n = 430)	
Full Time	122 (28.3)
Part Time	41 (9.5)
Unemployed	19 (4.4)
Unable to work due to illness/disability	97 (22.5)
Retired	134 (31.1)
Other	17 (3.9)
Finances (n = 420)	
Have enough or more than enough to make ends meet	331 (76.8)
Do not have enough to make ends meet	89 (20.6)
What symptom are you thinking about? (n = 462)*	
Pain	130 (28.1)
Respiratory symptom (e.g., shortness of breath, cough)	53 (11.5)
Mental health symptom (e.g., sadness, worry)	34 (7.4)
Fatigue	33 (7.1)
Gastrointestinal symptom (e.g., diarrhea, abdominal pain)	33 (7.1)
Abnormal blood sugar	23 (5)
Chest pain	17 (3.7)
Headache	16 (3.5)
Dizziness	13 (2.8)
Heart rate abnormalities (e.g., racing heart, palpitations)	9 (1.9)
Skin problem (e.g., rash, wound)	8 (1.7)
Physical limitation (e.g., difficulty walking)	7 (1.5)
Weakness	7 (1.5)
Difficulty sleeping	5 (1.1)
High blood pressure	5 (1.1)
Seizure	5 (1.1)
Other	64 (13.9)

*44 participants reported multiple symptoms

*Step 4: dimensionality & recalibration*

The 35 Self-Care Decisions Inventory items fit best into a 6-factor multidimensional structure in exploratory factor analysis (RMSEA = 0.05, CFI = 0.96, TLI = 0.94, and SRMR = 0.04). Velicer’s minimum average partial correlation and Horn’s parallel analysis confirmed the 6-factor structure (Additional File 1). Based on primary item loadings (Table 3) we identified six types of contextual factors that influence self-care decisions about symptoms – all significant factor loadings are presented.

**Table 3:** Self-Care Decisions Inventory item significant (p < 0.05) Geomin loadings and multidimensional structure

Self-care decisions inventory item	External	Urgency	Uncertainty	Cognitive/affective	Waiting/cue competition	Concealment
Others gave me advice	**0.839**					
Others helped me to make a decision	**0.784**					
Different people gave different advice about my symptom	**0.450**					
Someone else recognized the symptom before I did	**0.333**		0.232			0.292
I thought about decisions I made in the past when I had a similar symptom	0.215	**0.535**	0.261			
The symptom got worse suddenly		**0.672**				
When I had this symptom, I knew something was wrong		**0.611**			0.239	
The symptom was severe or bothersome		**0.659**		0.243		
I felt like something bad was going to happen		**0.430**	0.169		0.202	
I felt I needed to make a decision quickly		**0.407**			0.283	0.187
The symptom was different than what I expected		0.209	**0.522**			
It wasn’t clear to me what was causing the symptom			**0.555**			0.257
I didn’t know what the symptom meant		0.240	**0.739**			0.276
I thought the symptom might be due to something else	0.135		**0.580**			0.128
I wasn’t sure how important the symptom was			**0.580**		0.272	
When I had the symptom, I didn’t understand what was happening			**0.706**	0.217		
The symptom was new to me		0.171	**0.757**			
I recognized this symptom from the last time I had it			**0.711**			
The symptom was different than the last time I had it		0.232	**0.479**		0.225	
I felt too sad to make a decision				**0.716**		
My thinking was not clear so I could not make a decision				**0.780**		
I felt too anxious to make a decision				**0.751**		
I didn’t feel well enough to make a decision				**0.920**		
I felt too tired to make a decision				**0.808**	0.113	
I felt uncertain about what to do	0.102		0.237	**0.555**		
Other things were more important at the time					**0.506**	0.159
I thought I could wait to make a decision			0.141	0.272	**0.466**	
I felt that the symptom was nothing to worry about	0.175				**0.545**	
The symptom changed slowly	0.144	0.179			**0.225**	0.205
I thought I could tolerate the symptom					**0.760**	
Someone else needed my attention					**0.387**	0.237
I thought the symptom would go away on its own					**0.667**	
I felt embarrassed about my symptom	0.189					**0.510**
I didn’t want to burden my family		0.181				**0.463**
I didn’t want people to know about my symptom						**0.684**

Bolded factor loadings reflect items that preliminarily loaded onto the factor indicated in each column

Each represents a distinct and separately scored scale on the Self-Care Decisions Inventory. Scales were labeled ‘external,’ ‘urgency,’ ‘uncertainty,’ ‘cognitive/affective,’ ‘waiting/cue competition,’ and ‘concealment’ based on the initial literature review and the content of the items that significantly loaded onto that scale (Table 4). Correlations between scales ranged from 0.35 (urgency and uncertainty) to 0.13 (urgency and concealment).

**Table 4:** Interpretations of the six scales of the self-care decisions inventory

Scale	Interpretation
External	The extent to which input from other people influences self-care decision making. Higher scores indicate that self-care decision making is very influenced by the input of others
Urgency	The extent to which the perception of urgency or high stakes influences the patient’s self-care decision making. Higher scores indicate that the patient’s self-care decision making is very influenced by the perception that making a decision about what to do about the symptom is urgent or important
Uncertainty	The extent to which uncertainty or ambiguity, from incomplete information and/or difficulty interpreting the symptom, influence decision making. Higher scores indicate that the patient’s self-care decision making is very influenced by being unsure about the cause or meaning of the symptom
Cognitive/affective	The extent to which the patient’s thoughts or feelings influence decision making. Higher scores indicate that that patient’s thoughts and/or feelings interfere with or prevent decision making
Waiting/Cue competition	The extent to which situational factors delay decision making. Higher scores indicate that the patient is more likely to delay making a decision about their self-care because of competing priorities and/or a perception that the decision is not urgent
Concealment	The extent to which a desire to hide the symptom from others influences decision making. Higher scores indicate that the patient’s self-care decision making is very influenced by a desire to conceal the symptom from others

Each scale is a separate standardized score that can range from 0 to 100

**Fig. 3:**
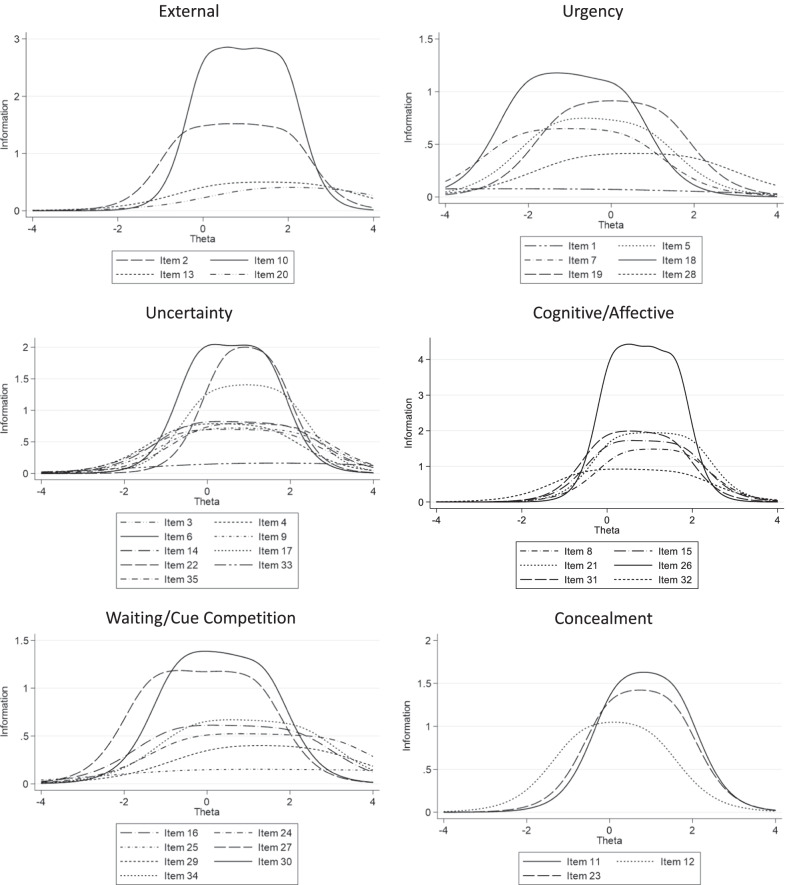
Self-care decisions inventory item information functions. Each pre-calibration item is shown within the six scales of the Self-Care Decisions Inventory. On the x-axis, theta represents the mean observed trait and the scale is standard errors around theta. On the y-axis, items providing more information about the trait with respect to greater discrimination have higher curves; items providing less information about the trait have lower curves, particularly those with a peak less than one

Four items were associated with the scale we labeled ‘external.’ Although all items were significant discriminators between low and high levels of external factors driving decision-making (Table 5), item 20 “*someone else recognized the symptom before I did*,” had the lowest value for discrimination, and provided the least information about the influence of external factors (Fig. 3). Further, based on category characteristic curves (Additional File 2), there had to be extremely high levels of the external influence (i.e. outside of the 95% confidence interval) for respondents to choose any response option above 1 (i.e. no influence). Therefore, item 20 was dropped from the ‘external’ scale.

**Table 5:** Scale-specific item discrimination and difficulty

	Discrimination within scale	Item difficultly	
*External*			
Others gave me advice	2.238 ± 0.284, *p* < 0.001		
		≥ 2	− 0.432 ± 0.083
		≥ 3	0.393 ± 0.078
		≥ 4	1.138 ± 0.106
		= 5	1.982 ± 0.171
Others helped me to make a decision	3.095 ± 0.570, *p* < 0.001		
		≥ 2	0.026 ± 0.068
		≥ 3	0.622 ± 0.077
		≥ 4	1.259 ± 0.109
		= 5	1.894 ± 0.160
Different people gave different advice about my symptom	1.255 ± 0.171, *p* < 0.001		
		≥ 2	0.305 ± 0.102
		≥ 3	0.956 ± 0.134
		≥ 4	1.900 ± 0.223
		= 5	2.695 ± 0.323
Someone else recognized the symptom before I did	1.134 ± 0.180, *p* < 0.001		
		≥ 2	1.105 ± 0.165
		≥ 3	1.594 ± 0.222
		≥ 4	2.197 ± 0.300
		= 5	3.169 ± 0.444
*Urgency*			
I thought about decisions I made in the past when I had a similar symptom	0.493 ± 0.115, *p* < 0.001		
		≥ 2	− 5.557 ± 1.277
		≥ 3	− 3.915 ± 0.896
		≥ 4	− 2.018 ± 0.486
		= 5	0.283 ± 0.218
The symptom got worse suddenly	1.536 ± 0.177, *p* < 0.001		
		≥ 2	− 1.479 ± 0.155
		≥ 3	− 0.917 ± 0.117
		≥ 4	− 0.251 ± 0.090
		= 5	0.743 ± 0.109
When I had this symptom, I knew something was wrong	1.434 ± 0.168, *p* < 0.001		
		≥ 2	− 2.272 ± 0.230
		≥ 3	− 1.405 ± 0.154
		≥ 4	− 0.545 ± 0.102
		= 5	0.384 ± 0.099
The symptom was severe or bothersome	1.941 ± 0.228, *p* < 0.001		
		≥ 2	− 2.105 ± 0.190
		≥ 3	− 1.437 ± 0.135
		≥ 4	− 0.743 ± 0.095
		= 5	0.226 ± 0.082
I felt like something bad was going to happen	1.700 ± 0.199, *p* < 0.001		
		≥ 2	− 1.114 ± 0.124
		≥ 3	− 0.278 ± 0.086
		≥ 4	0.332 ± 0.088
		= 5	1.186 ± 0.127
I felt I needed to make a decision quickly	1.139 ± 0.149, *p* < 0.001		
		≥ 2	− 0.855 ± 0.140
		≥ 3	0.068 ± 0.106
		≥ 4	1.100 ± 0.157
		= 5	1.978 ± 0.242
*Uncertainty*			
The symptom was different than what I expected	1.492 ± 0.150, *p* < 0.001		
		≥ 2	− 0.652 ± 0.106
		≥ 3	0.021 ± 0.089
		≥ 4	0.971 ± 0.115
		= 5	1.939 ± 0.184
It wasn’t clear to me what was causing the symptom	1.575 ± 0.159, *p* < 0.001		
		≥ 2	− 0.690 ± 0.104
		≥ 3	− 0.079 ± 0.087
		≥ 4	0.675 ± 0.098
		= 5	1.492 ± 0.145
I didn’t know what the symptom meant	2.575 ± 0.255, *p* < 0.001		
		≥ 2	− 0.254 ± 0.075
		≥ 3	0.197 ± 0.071
		≥ 4	0.863 ± 0.084
		= 5	1.431 ± 0.115
I thought the symptom might be due to something else	1.509 ± 0.155, *p* < 0.001		
		≥ 2	− 0.365 ± 0.095
		≥ 3	0.327 ± 0.091
		≥ 4	1.164 ± 0.126
		= 5	2.156 ± 0.205
I wasn’t sure how important the symptom was	1.621 ± 0.161, *p* < 0.001		
		≥ 2	− 0.508 ± 0.096
		≥ 3	0.107 ± 0.085
		≥ 4	1.007 ± 0.112
		= 5	2.001 ± 0.185
When I had the symptom, I didn’t understand what was happening	2.107 ± 0.210, *p* < 0.001		
		≥ 2	− 0.026 ± 0.078
		≥ 3	0.667 ± 0.084
		≥ 4	1.190 ± 0.108
		= 5	1.846 ± 0.156
The symptom was new to me	2.505 ± 0.278, *p* < 0.001		
		≥ 2	0.357 ± 0.073
		≥ 3	0.677 ± 0.079
		≥ 4	1.082 ± 0.096
		= 5	1.545 ± 0.126
I recognized this symptom from the last time I had it	− 0.717 ± 0.118, *p* < 0.001		
		≥ 2	3.159 ± 0.515
		≥ 3	2.482 ± 0.408
		≥ 4	1.506 ± 0.268
		= 5	0.017 ± 0.149
The symptom was different than the last time I had it	1.582 ± 0.166, *p* < 0.001		
		≥ 2	− 0.107 ± 0.089
		≥ 3	0.537 ± 0.094
		≥ 4	1.313 ± 0.135
		= 5	2.235 ± 0.213
*Cognitive/affective*			
I felt too sad to make a decision	2.168 ± 0.226, *p* < 0.001		
		≥ 2	0.303 ± 0.076
		≥ 3	0.777 ± 0.087
		≥ 4	1.392 ± 0.121
		= 5	1.851 ± 0.157
My thinking was not clear so I could not make a decision	2.352 ± 0.230, *p* < 0.001		
		≥ 2	0.007 ± 0.074
		≥ 3	0.484 ± 0.076
		≥ 4	1.113 ± 0.098
		= 5	1.778 ± 0.141
I felt too anxious to make a decision	2.504 ± 0.254, *p* < 0.001		
		≥ 2	0.147 ± 0.072
		≥ 3	0.750 ± 0.081
		≥ 4	1.334 ± 0.109
		= 5	1.962 ± 0.158
I didn’t feel well enough to make a decision	3.840 ± 0.445, *p* < 0.001		
		≥ 2	0.099 ± 0.065
		≥ 3	0.556 ± 0.067
		≥ 4	1.053 ± 0.082
		= 5	1.599 ± 0.112
I felt too tired to make a decision	2.513 ± 0.241, *p* < 0.001		
		≥ 2	− 0.112 ± 0.073
		≥ 3	0.390 ± 0.073
		≥ 4	0.862 ± 0.084
		= 5	1.505 ± 0.119
I felt uncertain about what to do	1.713 ± 0.164, *p* < 0.001		
		≥ 2	− 0.564 ± 0.095
		≥ 3	0.070 ± 0.083
		≥ 4	0.794 ± 0.098
		= 5	1.732 ± 0.154
*Waiting/cue competition*			
I thought I could wait to make a decision	1.400 ± 0.153, *p* < 0.001		
		≥ 2	− 0.889 ± 0.122
		≥ 3	− 0.057 ± 0.093
		≥ 4	0.966 ± 0.121
		= 5	2.122 ± 0.214
I felt that the symptom was nothing to worry about	1.297 ± 0.153, *p* < 0.001		
		≥ 2	− 0.470 ± 0.109
		≥ 3	0.579 ± 0.109
		≥ 4	1.670 ± 0.186
		= 5	3.001 ± 0.341
The symptom changed slowly	0.695 ± 0.120, *p* < 0.001		
		≥ 2	− 0.699 ± 0.191
		≥ 3	0.642 ± 0.181
		≥ 4	2.192 ± 0.380
		= 5	4.614 ± 0.800
I thought I could tolerate the symptom	1.971 ± 0.212, *p* < 0.001		
		≥ 2	− 1.360 ± 0.127
		≥ 3	− 0.593 ± 0.089
		≥ 4	0.309 ± 0.081
		= 5	1.167 ± 0.113
Someone else needed my attention	1.120 ± 0.155, *p* < 0.001		
		≥ 2	0.153 ± 0.107
		≥ 3	0.769 ± 0.133
		≥ 4	1.620 ± 0.212
		= 5	2.637 ± 0.334
I thought the symptom would go away on its own	2.105 ± 0.231, *p* < 0.001		
		≥ 2	− 0.725 ± 0.094
		≥ 3	− 0.166 ± 0.078
		≥ 4	0.488 ± 0.082
		= 5	1.332 ± 0.120
Other things were more important at the time	1.458 ± 0.171, *p* < 0.001		
		≥ 2	− 0.392 ± 0.100
		≥ 3	0.316 ± 0.093
		≥ 4	1.178 ± 0.135
		= 5	2.259 ± 0.234
*Concealment*			
I felt embarrassed about my symptom	2.260 ± 0.333, *p* < 0.001		
		≥ 2	0.137 ± 0.075
		≥ 3	0.631 ± 0.086
		≥ 4	1.028 ± 0.105
		= 5	1.581 ± 0.145
I didn’t want to burden my family	1.810 ± 0.225, *p* < 0.001		
		≥ 2	− 0.710 ± 0.101
		≥ 3	− 0.147 ± 0.083
		≥ 4	0.335 ± 0.085
		= 5	0.957 ± 0.111
I didn’t want people to know about my symptom	2.112 ± 0.296, *p* < 0.001		
		≥ 2	− 0.036 ± 0.078
		≥ 3	0.495 ± 0.083
		≥ 4	0.977 ± 0.105
		= 5	1.528 ± 0.144

Six items were associated with the scale we labeled ‘urgency,’ All items were significant discriminators between low and high levels of urgency (Table 5); but item 1, “*I thought about decisions I made in the past when I had a similar symptom*,” had the lowest value for discrimination and not all response options discriminated significantly. In addition, item 1 provided almost no information about the influence of urgency (Fig. 3), and there was a very low threshold for higher probability of respondents choosing higher response options. Therefore, item 1 was dropped from the ‘urgency’ scale.

Nine items loaded on the scale we labeled ‘uncertainty.’ All items were significant discriminators between low and high levels of uncertainty (Table 5); however, there were redundancies with respect to item information, especially involving these items: item 3 “*The symptom was different than what I expected*,” and item 35 “*The symptom was different than the last time I had it*” (Fig. 3). Additionally, item 33 “*I recognized this symptom from the last time I had it*” was the weakest discriminator and provided the least information about uncertainty. Items 3, 33 and 35 were omitted from the ‘uncertainty’ scale.

Six items were associated with the scale we labeled ‘cognitive/affective.’ All six items discriminated significantly (Table 5). However, for item 32 “*I felt uncertain about what to do*”, not all response options were significant discriminators (Additional File 2) and item 32 also provided the least information about the influence of the individual’s cognitive/affective state (Fig. 3). Accordingly, item 32 was dropped from the ‘cognitive/affective’ scale.

Seven items loaded on the scale we labeled ‘waiting/cue competition.’ All items discriminated significantly between low and high levels of waiting/cue competition (Table 5). However, items 25 “*The symptom changed slowly*” and 29 “*Someone else needed my attention”* had the lowest values for discrimination and provided the least information about the ‘waiting/cue competition’ scale (Fig. 3). Hence, items 25 and 29 were omitted from the ‘waiting/cue competition’ scale.

Finally, three items loaded on the scale we labeled ‘concealment.’ All three items were significant discriminators between low and high levels of concealment (Table 5) and all items provided sufficient information about concealment (Fig. 3). Accordingly, all three items were retained in the ‘concealment’ scale.

*Step 5: construct validity*

Correlations between the six new Self-Care Decisions Inventory scales and the four domains of the Melbourne DMQ were tested (Table 6).

**Table 6:** Convergent Validity Testing with Melbourne Decision-Making Questionnaire Domains

	External	Urgency	Uncertainty	Cognitive/affective	Waiting/cue competition	Concealment
Vigilance	–	–	–	–	–	–
Buck passing	0.211	–	–	0.363	0.170	0.233
Procrastination	–	–	0.178	0.402	0.239	0.266
Hypervigilance	0.185	–	0.160	0.427	–	0.312

Values shown are significant (*p* < 0.05) linear correlations with Bonferroni correction applied

The Self-Care Decisions Inventory external scale was modestly associated with buck passing and hypervigilance. The Self-Care Decisions Inventory uncertainty scale was modestly associated with procrastination and hypervigilance. The Self-Care Decisions Inventory cognitive/affective scale was associated with buck passing, procrastination, and hypervigilance. The Self-Care Decisions Inventory waiting/cue competition scale was associated modestly with buck passing and procrastination. The Self-Care Decisions Inventory concealment scale was associated with buck passing, procrastination and hypervigilance. No scale on the Self-Care Decisions Inventory was significantly associated with the Melbourne DMQ vigilance domain, and the Self-Care Decisions Inventory urgency scale was not associated with any Melbourne DMQ domain.

We also evaluated differences in scale scores of the Self-Care Decisions Inventory between individuals with adequate and inadequate self-care management (Table 7). Adequate self-care management is defined as a score ≥ 70 on the SC-CII Management Scale [24].

**Table 7:** Criterion validity testing comparing the six scales of the self-care decisions inventory with adequate versus inadequate self-care management

	Adequate self-care management^a^ (n = 140) mean (sd)	Inadequate Self-care management^a^ (n = 289) mean (sd)	*t*-statistic	Effect size *Hedge’s g*
External	31.56 (26.89)	23.76 (22.35)	− 2.97 (*p* = 0.003)	0.32
Urgency	65.14 (20.54)	55.40 (24.02)	− 4.34 (*p* < 0.001)	0.42
Uncertainty	37.29 (24.16)	30.98 (25.24)	− 2.49 (*p* = 0.013)	0.25
Cognitive/affective	25.54 (25.42)	26.22 (26.52)	0.25 (*p* = 0.8)	0.03
Waiting/cue competition	39.82 (23.01)	40.14 (23.23)	0.13 (*p* = 0.89)	0.01
Concealment	36.75 (31.39)	34.00 (28.53)	− 0.88 (*p* = 0.38)	0.09

^a^Adequate self-care management is defined as a score ≥ 70 on the SC-CII Management Scale

There were statistically significant differences in the scores on the external, urgency, and uncertainty scales ranging from small to medium effect sizes. This partially supported our hypothesis that the scales of the Self-Care Decisions Inventory would correlate with adequate self-care. Individuals with higher urgency had statistically significantly higher self-care management, as hypothesized. However, those with higher uncertainty also had higher self-care management scores.

*Step 6: precision and reliability*

**Fig. 4:**
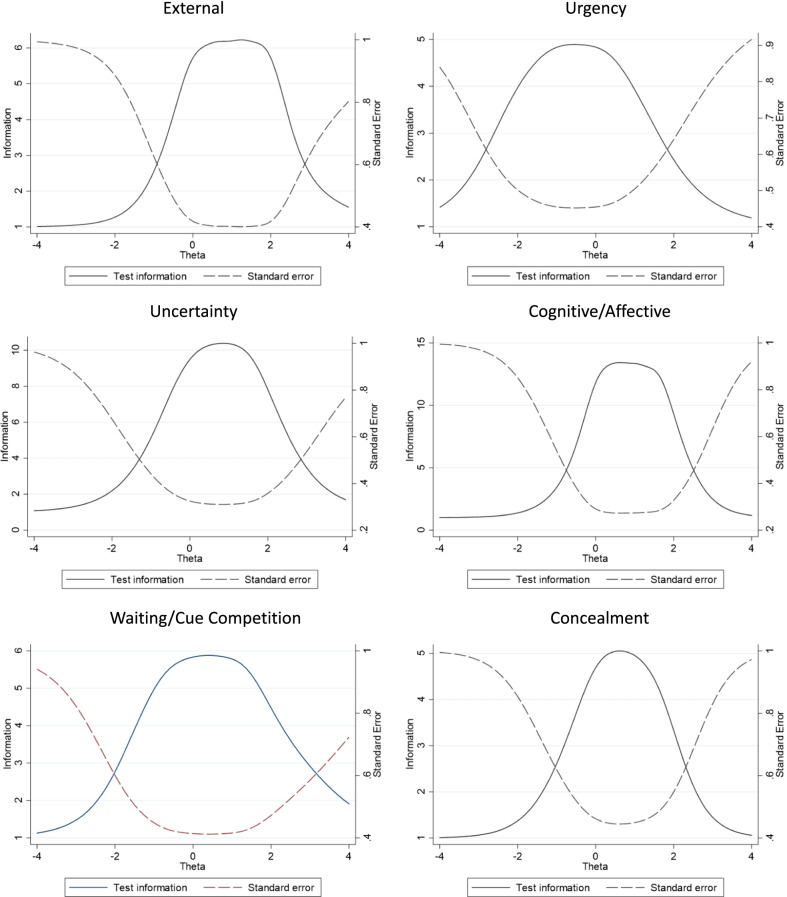
Recalibrated test information functions for each scale of the Self-Care Decisions Inventory. Each post-calibration scale of the Self-Care Decisions Inventory is presented regarding the degree to which the factor items collectively inform the trait (left y-axis—information), and range of underlying trait (x-axis with theta representing the mean observed trait and the scale is standard errors around theta) where the scale is most precise (right y-axis – standard error)

Using IRT, test information function graphs along with plotted standard errors inform the range of underlying contextual factor where the scale is most precise; these data are provided in Fig. 4. Using confirmatory factor analysis with recalibrated domains, multidimensional reliability (i.e., factor determinacy score) was high at 0.86.

*Step 7: differential item functioning*

No significant uniform or non-uniform differential item functioning was detected by self-identified gender.

### Scoring and reference ranges

**Fig. 5:**
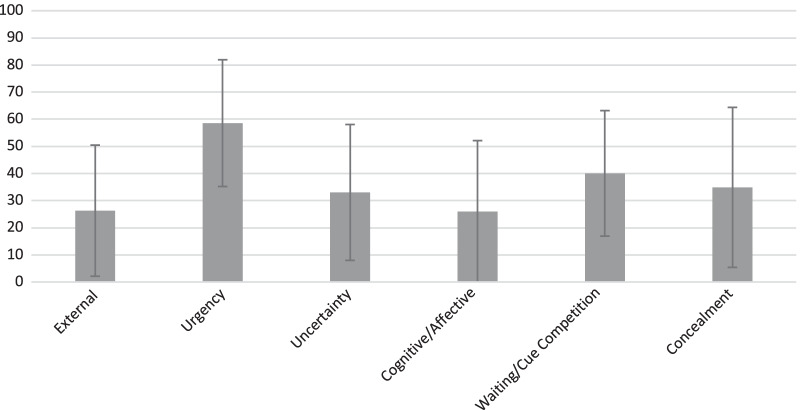
Standardized scores on the Self-Care Decisions Inventory. The mean and standard deviation of the standardized scores for each scale of the Self-Care Decisions Inventory in the current sample are displayed

Separate standardized scoring (fixed score range from 0 to 100) is recommended for the six scales of the Self-Care Decisions Inventory. There is no total score. Mean ± standard deviation of standardized scores were external (26.30 ± 24.28), urgency (58.57 ± 25.38), uncertainty (33.03 ± 25.04), cognitive/affective (26.00 ± 26.04), waiting/cue competition (40.04 ± 23.13), and concealment (34.89 ± 29.48) in this derivation sample (Fig. 5).

## Discussion

The Self-Care Decisions Inventory is a 27-item self-report instrument measuring contextual factors influencing self-care decisions about symptoms with six scales: ‘external,’ ‘urgency,’ ‘uncertainty,’ ‘cognitive/affective,’ ‘waiting/cue competition,’ and ‘concealment.’ To our knowledge, this is the first instrument to operationalize naturalistic decision making to measure the contextual factors that influence self-care decisions.

A core premise of naturalistic decision making is that decisions take place in real-world environments that are dynamically evolving [2]. As such, decisions are often made with incomplete information. The ‘uncertainty’ scale assesses uncertainty that arises from ambiguity about the cause or meaning of a symptom. Situational factors also influence decision making and the ‘urgency’ scale measures the influence of feeling that the response to a symptom is time sensitive. The ‘waiting/cue competition’ scale assesses the influence of competing priorities. Together, these three scales (uncertainty, urgency, and waiting/cue competition) provide insight into how patients use information about their symptoms to make decisions. For example, a patient with a high uncertainty score may need support in learning how to assess the severity of their symptoms.

The involvement of multiple individuals (e.g., family, clinicians) may enhance or complicate decision-making. Individuals who score high on the ‘external’ scale are influenced strongly by the input of others. In The Theory of Dyadic Illness Management, the relationship between patients and their care partners is transactional and interdependent as they navigate the patient’s illness together [27]. Decision-making is a dyadic management behavior and there is variability in how patients and their care partners collaborate to make decisions. Prior studies have shown that indeed self-care is a dyadic phenomenon in chronic illness; [28, 29] but the dyadic nature of decision-making in response to symptoms is unknown. Further research on caregiver contributions to self-care and dyadic decision-making about symptoms is needed to better understand how patients and their care partners collaborate to manage symptoms of chronic illness. Some adults with chronic illness may instead wish to hide their symptoms from others. The ‘concealment’ scale measures this concept by assessing the extent to which a desire to hide symptoms influences decision making.

The initial draft of the instrument included several items related to prior experience, thought theoretically to inform the assessment of the situation and decision choices. Interestingly, the prior experience items discriminated well between respondents at the extremes (i.e., prior experience having no influence or much influence), but intermediate response options did not discriminate well, and the items were eliminated during recalibration. Respondents to our cognitive interviews universally endorsed prior experience. This is similar to our previous findings in adults with heart failure who reported that prior experience was valuable in improving their ability to recognize and interpret symptoms [3]. A lack of prior experience is reflected in the ‘uncertainty’ scale.

One’s cognitive or affective state at the time when a symptom occurs also influences decision making, a concept measured by the ‘cognitive/ affective’ scale. In this study. individuals who were highly influenced by thoughts or feelings (i.e., higher score on the cognitive/affective scale) had decisional coping styles that were more maladaptive. Indeed, the contextual factors measured by the Self-Care Decisions Inventory can complement assessment of coping with decisional conflict. The Melbourne DMQ [7] pattern of hypervigilance was modestly associated with the external and uncertainty scales, which could suggest that, for some, the input of others and incomplete information leads to a chaotic coping pattern. The concealment scale, which correlated with the Melbourne DMQ patterns of hypervigilance, buck passing, and procrastination, could also be seen as a coping response. The urgency scale was not associated with any coping patterns on the Melbourne DMQ. Perhaps urgency caused by a symptom that is severe or worsening leads to a swift decision rather than decisional conflict. Investigators who are interested in the contextual factors derived from the naturalistic decision making framework and also want to understand how people cope with decisional conflict may want to use both instruments in future research.

Several of the contextual factors measured by this new instrument appear to be amenable to interventions to improve decision-making about symptoms, which may improve self-care. In this study, the influence of external factors, urgency, and uncertainty differed significantly between those with (SC-CII management scale score ≥ 70) and without adequate self-care management. These results confirm findings from other studies that the perception of urgency and importance prompts engagement in self-care [30]. Surprisingly, there was more uncertainty in those with adequate self-care management compared with those with inadequate self-care management. This difference may be explained by considering that the self-care management scale measures responses to symptoms that include calling the provider for guidance. People may be more tempted to call the provider if they feel uncertain about what to do when they have a symptom. Finally, those with adequate self-care were more influenced by the input of others (external scale). This suggests that those with adequate self-care management are more likely to consult with others (e.g., family, clinicians) when making decisions about what to do about symptoms. Patients may be differentially influenced by contextual factors based on the severity of the condition, whether the condition is life-limiting, and social stigma surrounding it. Future research might compare decision-making between groups of individuals with different chronic conditions to gain insights that could inform tailored self-care decision-making interventions.

Limitations include a convenience sample that was predominantly female, White, and residing in the United States. The first five interviewees were women, but content validity was later assessed by a more representative group of two men and three women. All data were cross-sectional. Our response rate was low (38.2%), which is common in online surveys [31]. Since the invited participants were anonymous, we are unable to assess if there were significant differences between those who completed the survey and those who did not, which might have biased our sample. Further testing in more diverse populations is needed to ensure generalizability to all adults with symptomatic chronic illness. Based on simulation studies for IRT models [32, 33], for this 27-item instrument we recommend enrolling a minimum of 500 participants in future studies. We did not evaluate test–retest reliability, so stability of the decision-making pattern(s) is unknown. Although some aspects of decision-making are likely trait-like and stable across contexts [34], naturalistic decision-making is situation specific and variable. Short-term stability should be tested in future research. Finally, responses to many of the questions indicate that 5 response options may not be ideal or even necessary; the lack of significant differential item functioning by gender also will need to be confirmed in future studies. After additional validation, future refinements of the instrument may include limiting response options or even dichotomizing responses.

## Conclusion

The 27-item Self-Care Decisions Inventory is a new instrument developed with input from patients, clinicians, and researchers. It measures six contextual factors that influence everyday decision-making about symptoms of chronic illness. Content validity is excellent and the instrument has high multidimensional reliability. While additional testing is indicated, initial psychometric analysis indicates that the Self-Care Decisions Inventory may be useful in research to better understand the processes that persons use to make decisions about their symptoms.

## Supplementary Information

**Additional file 1:** Velicer’s minimum average partial (MAP) correlation, Horn's output, and Horn’s parallel analysis graph.

**Additional file 2:** Category characteristic curves for each item.

## Data Availability

The datasets used and/or analyzed during the current study are available from the corresponding author on reasonable request.

## Abbreviations

CFI: Comparative fit index
COSMIN: COnsensus-based standards for the selection of health measurement instruments
CVI: Content validity index
I-CVI: Item content validity index
IRT: Item response theory
FIML: Full information maximum likelihood estimation
Melbourne DMQ: Melbourne decision making questionnaire
RMSEA: Root mean square error of approximation
SC-CII: Self-care of chronic illness inventory
S-CVI: Scale content validity index
SRMR: Standardized root mean square residual
TLI: Tucker-Lewis index
