# Supplementary material for: Development and testing of an instrument to measure contextual factors influencing self-care decisions among adults with chronic illness
Source: Health Qual Life Outcomes. 2022 May 23;20:83. doi: 10.1186/s12955-022-01990-2 (PMC9125861; doi:10.1186/s12955-022-01990-2)
Supplement: Supplementary file 1 — Additional file 1: Velicer’s minimum average partial (MAP) correlation, Horn's output, and Horn’s parallel analysis graph. [file 12955_2022_1990_MOESM1_ESM.pdf]

Minimum Average Partial Correlation for Number of Principal Components
NOTE: Pick number of components (m) at which fm is minimum. If f1 > f0 (average intervariable correlation) then no components should be extracted.

m = 0	f0 =	.05481562
m = 1	f1 =	.02031914
m = 2	f2 =	.0155259
m = 3	f3 =	.01218861
m = 4	f4 =	.01156191
m = 5	f5 =	.01150647
m = 6	f6 =	.01107013
m = 7	f7 =	.01235798
m = 8	f8 =	.01338821
m = 9	f9 =	.01478484
m = 10	f10 =	.01651844
m = 11	f11 =	.01811712
m = 12	f12 =	.02016466
m = 13	f13 =	.02235991
m = 14	f14 =	.02473236
m = 15	f15 =	.02745828
m = 16	f16 =	.03029986
m = 17	f17 =	.03357179
m = 18	f18 =	.0366319
m = 19	f19 =	.04109616
m = 20	f20 =	.04594404
m = 21	f21 =	.05188819
m = 22	f22 =	.05825501
m = 23	f23 =	.06536314
m = 24	f24 =	.07367909
m = 25	f25 =	.08481699
m = 26	f26 =	.09956326
m = 27	f27 =	.11600291
m = 28	f28 =	.1368907
m = 29	f29 =	.15809474
m = 30	f30 =	.19757832
m = 31	f31 =	.25895152
m = 32	f32 =	.33943009
m = 33	f33 =	.53548909
m = 34	f34 =	1

minap procedure suggests that 6 principal components should be extracted

Results of Horn's Parallel Analysis for principal components
1050 iterations, using the mean estimate

Component or Factor	Adjusted Eigenvalue	Unadjusted Eigenvalue	Estimated Bias
1	7.5489817	8.0727692	.5237875
2	2.5630657	2.9985336	.43546796
3	2.2707573	2.6784854	.40772808
4	1.5597081	1.8998093	.34010124
5	1.214481	1.5247516	.31027055
6	1.1135753	1.3916951	.2781198
7	.91723048	1.1828252	.26559472
8	.75528898	1.0026479	.24735892

Criterion: retain adjusted components > 1

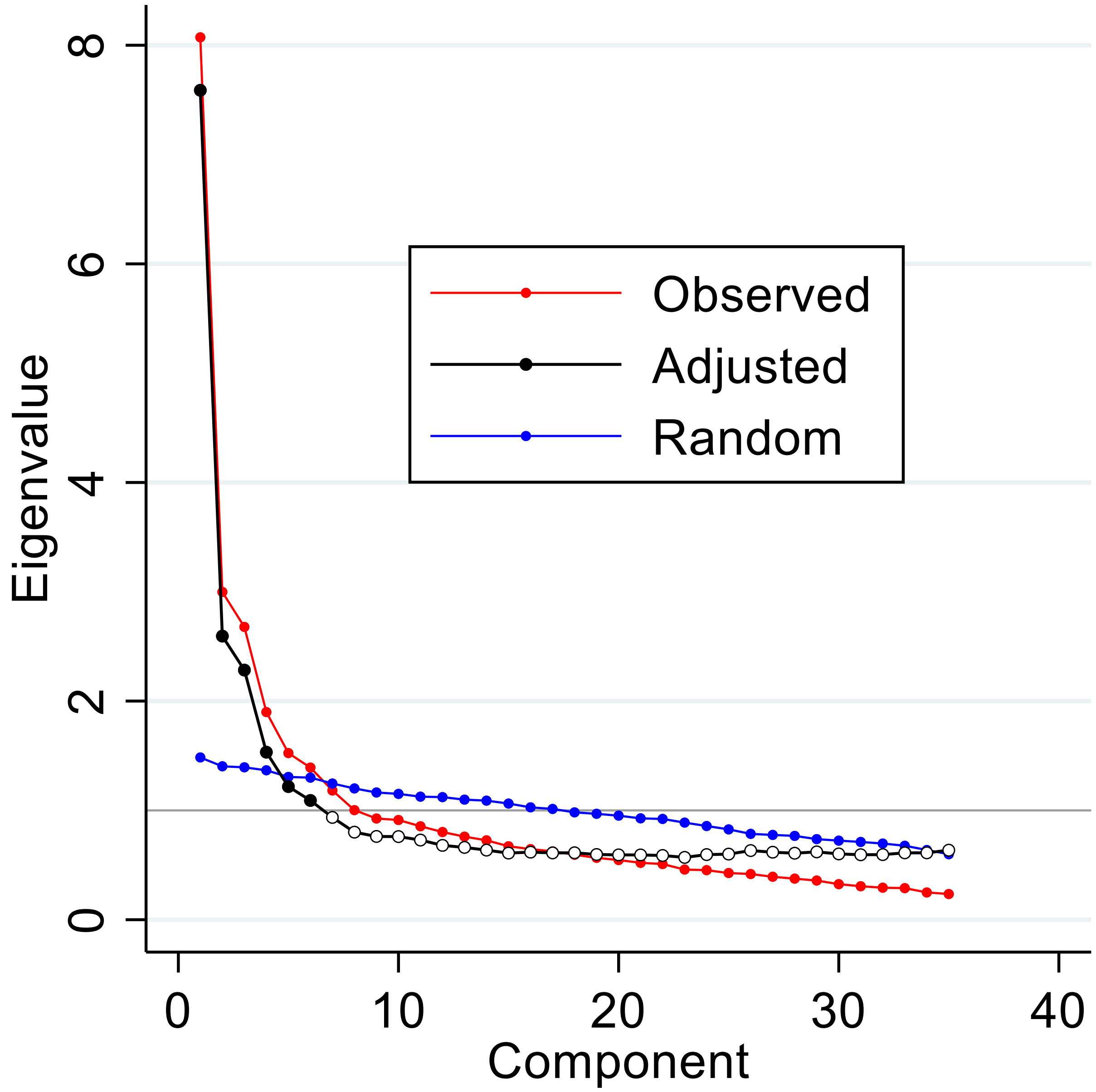
Observed
Adjusted
Random
Eigenvalue
0
2
4
6
8
Component
0
10
20
30
40
