# Supplementary material for: Development and testing of an instrument to measure contextual factors influencing self-care decisions among adults with chronic illness
Source: Health Qual Life Outcomes. 2022 May 23;20:83. doi: 10.1186/s12955-022-01990-2 (PMC9125861; doi:10.1186/s12955-022-01990-2)

Category Characteristic Curves for Each Item

Category characteristic curves show the probability (y-axis) of the respondent selecting the response option (1-5) at varying levels of the underlying trait (x-axis). Items are organized by scale. Items in red were removed from the Self-Care Decisions Scale during recalibration.

<u>External Scale</u>

Item 2. Others gave me advice

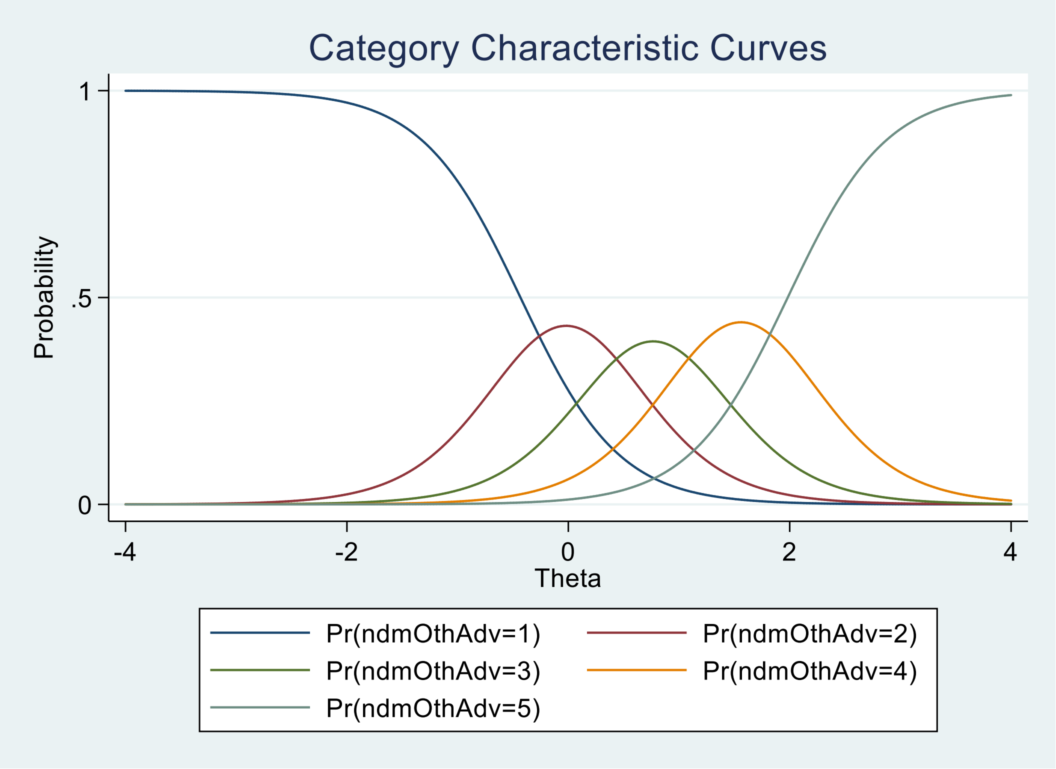

Item 10. Others helped me to make a decision

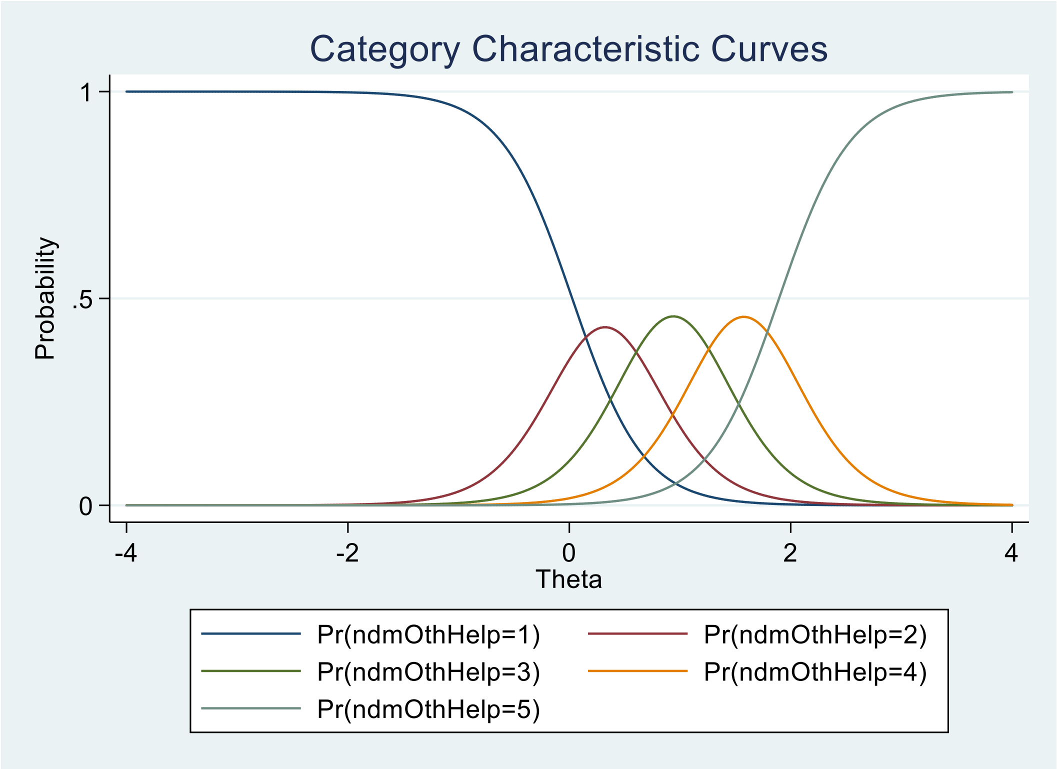

Item 13. Different people gave different advice about my symptom

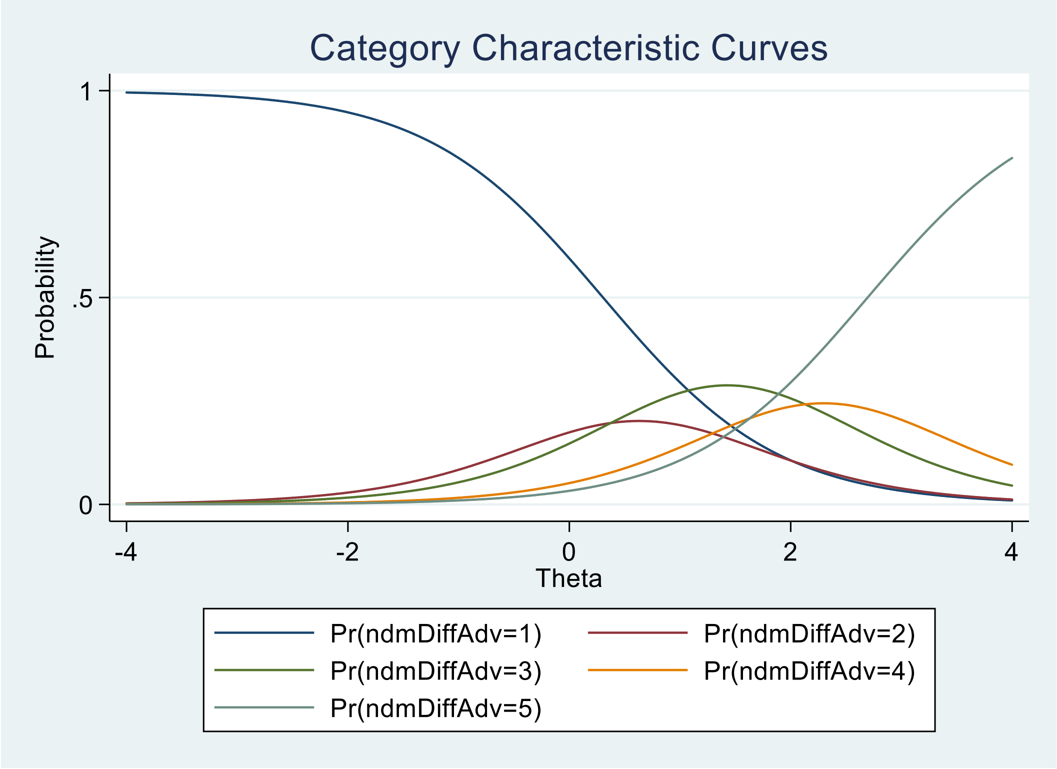

Item 20. Someone else recognized the symptom before I did

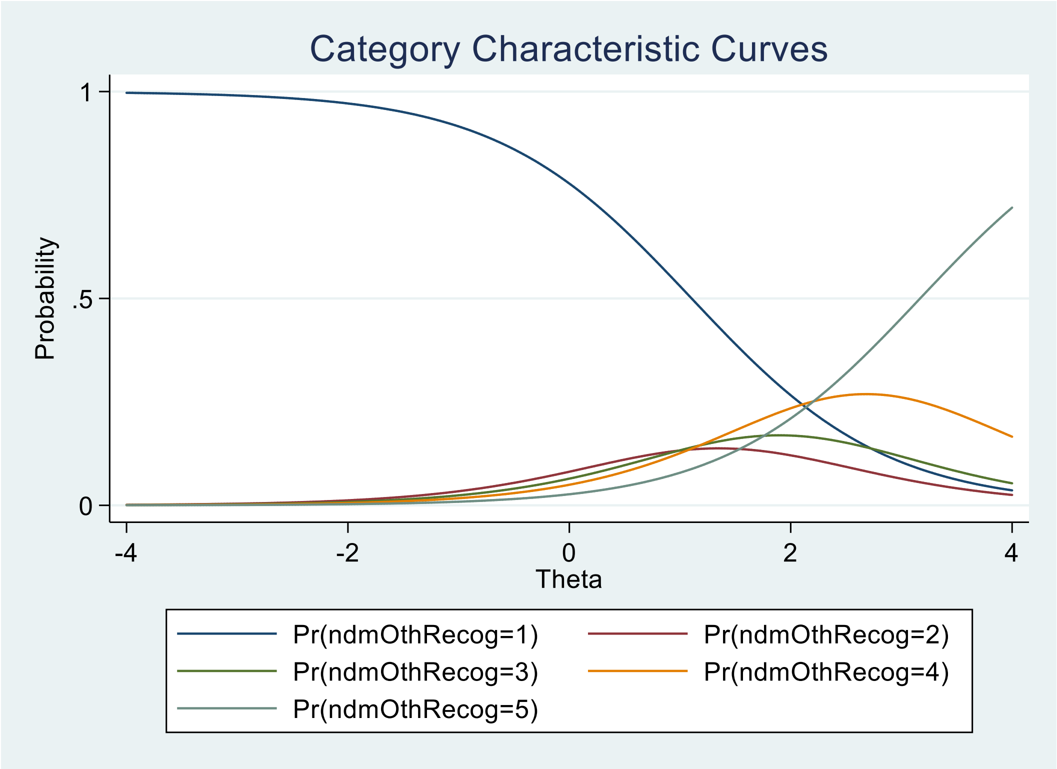

Urgency Scale

Item 1. I thought about decisions I made in the past when I had a similar symptom

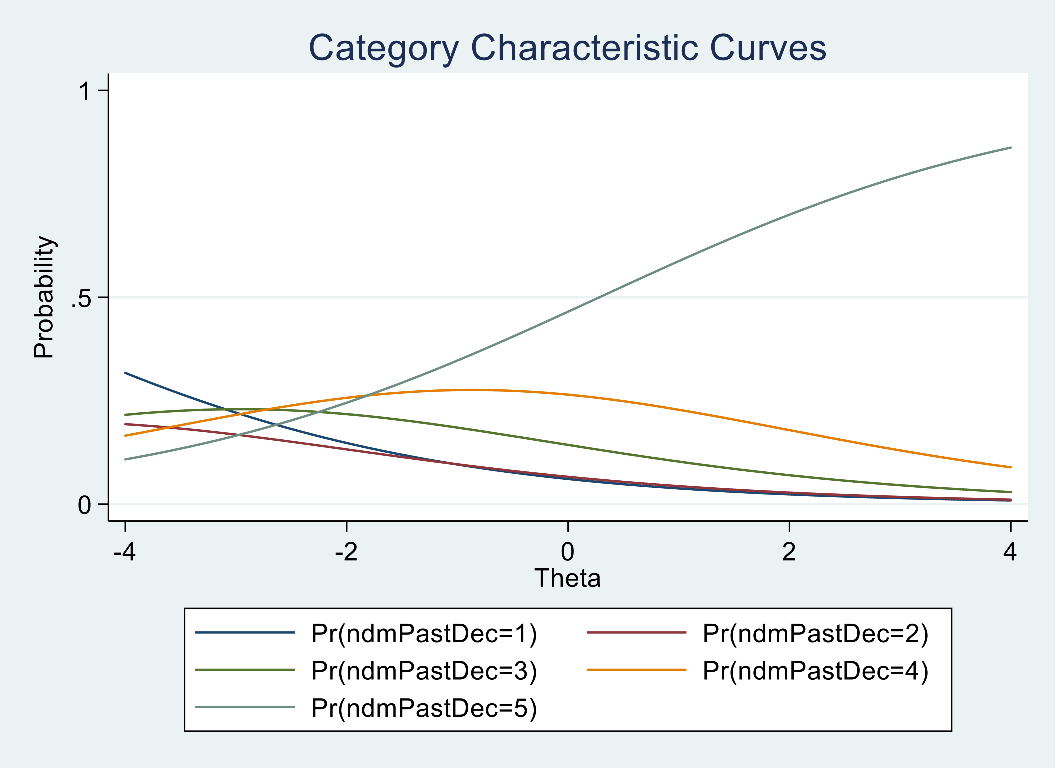

Item 5. The symptom got worse suddenly

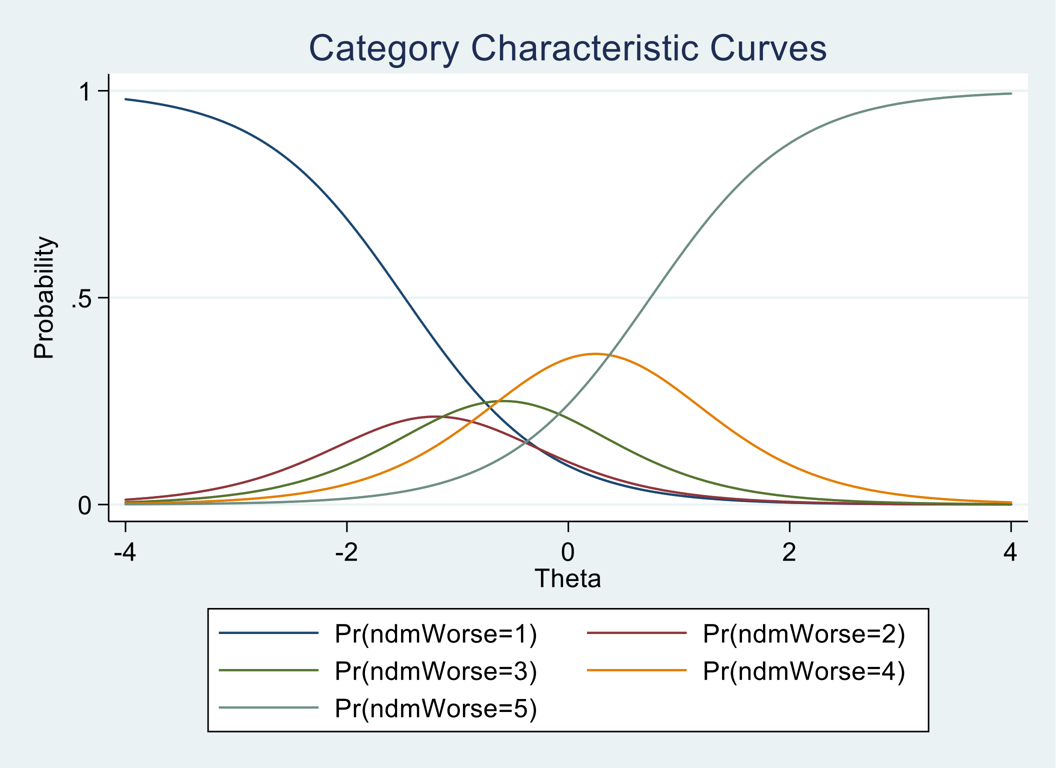

Item 7. When I had the symptom I knew something was wrong

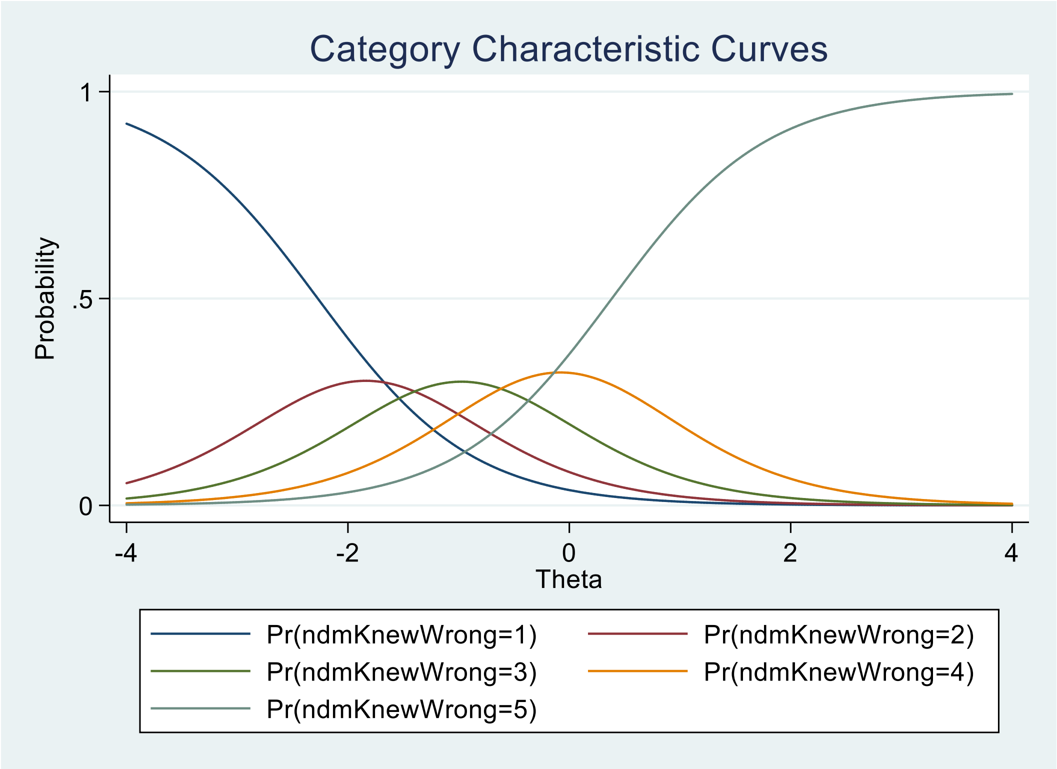

Item 18. The symptom was severe or bothersome

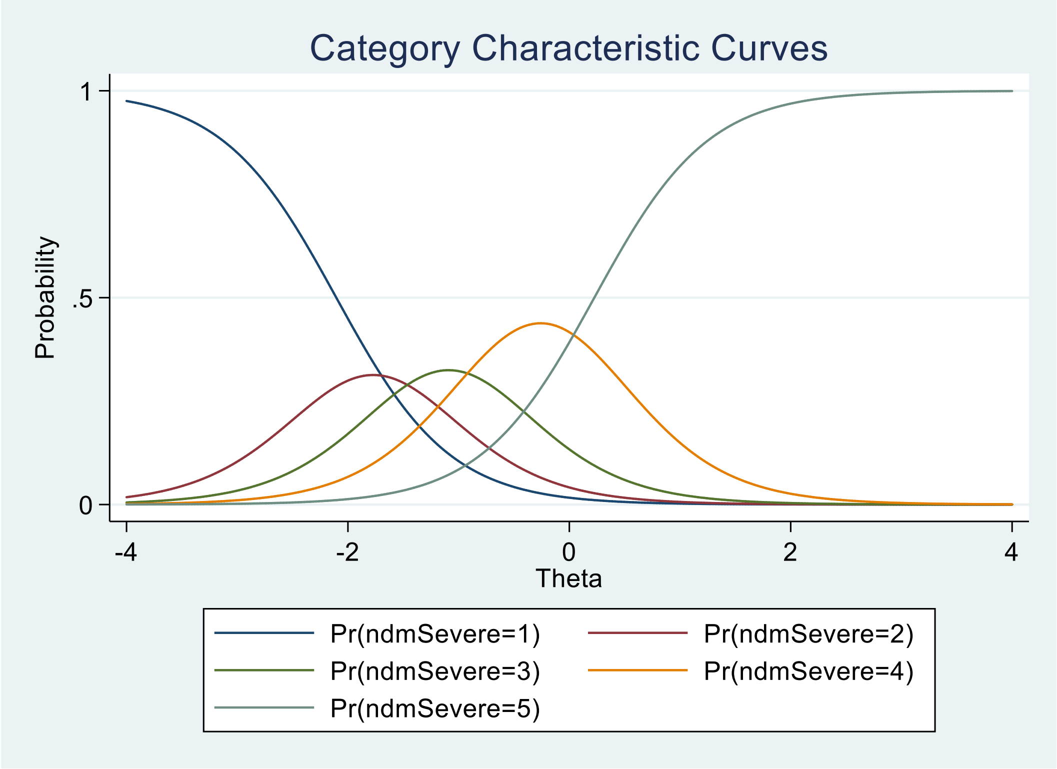

Item 19. I felt like something bad was going to happen

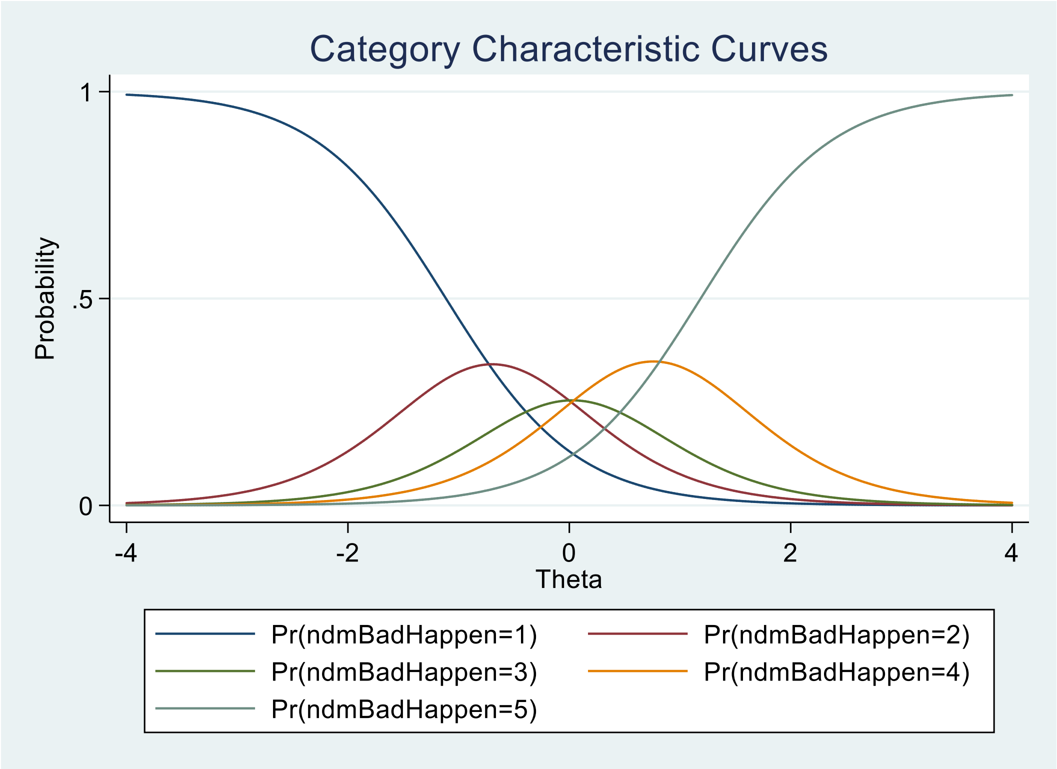

Item 28. I felt I needed to make a decision quickly

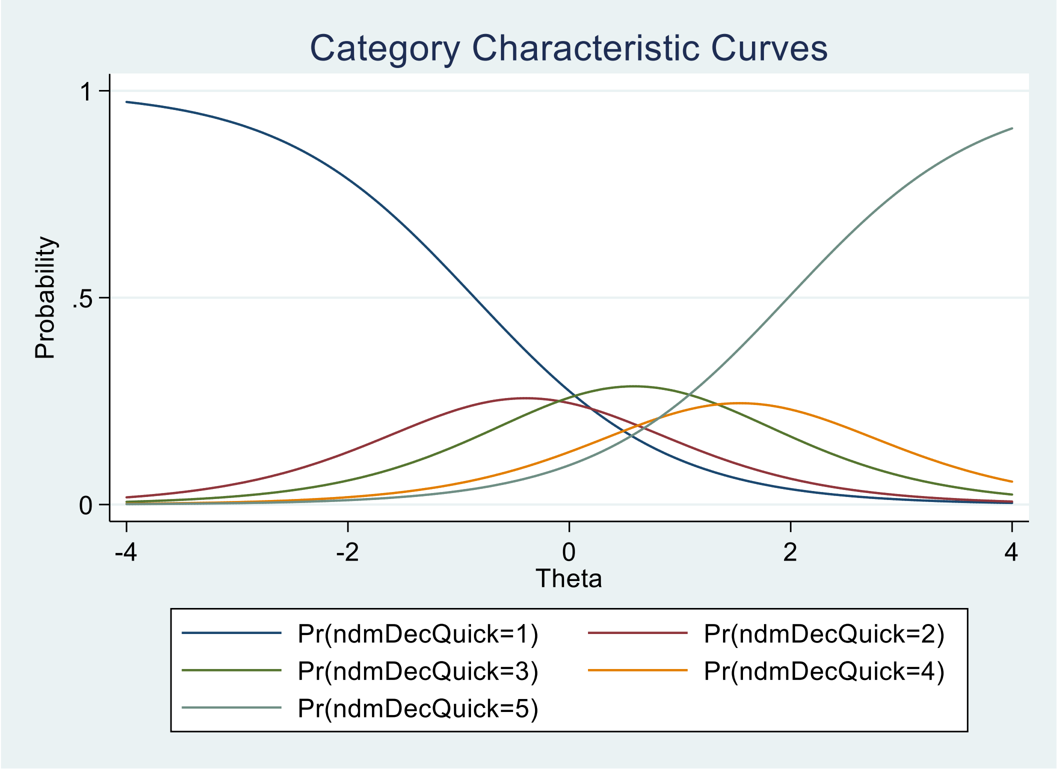

Uncertainty Scale

Item 3. The symptom was different than what I expected

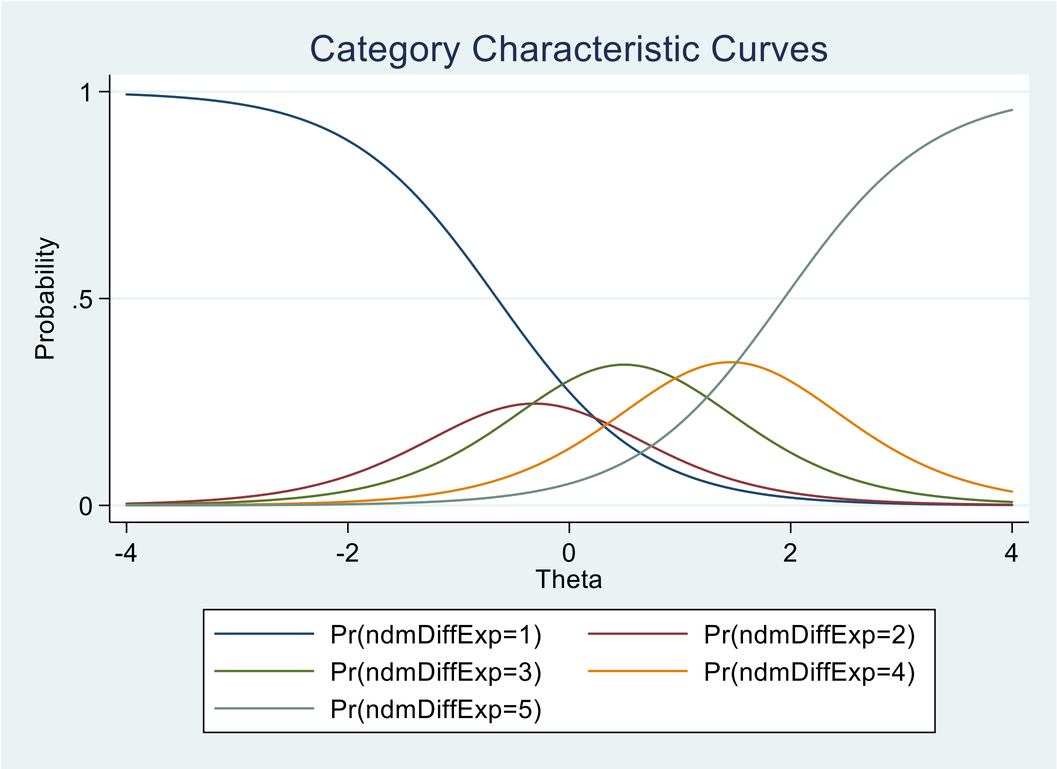

Item 4. It wasn't clear to me what was causing the symptom

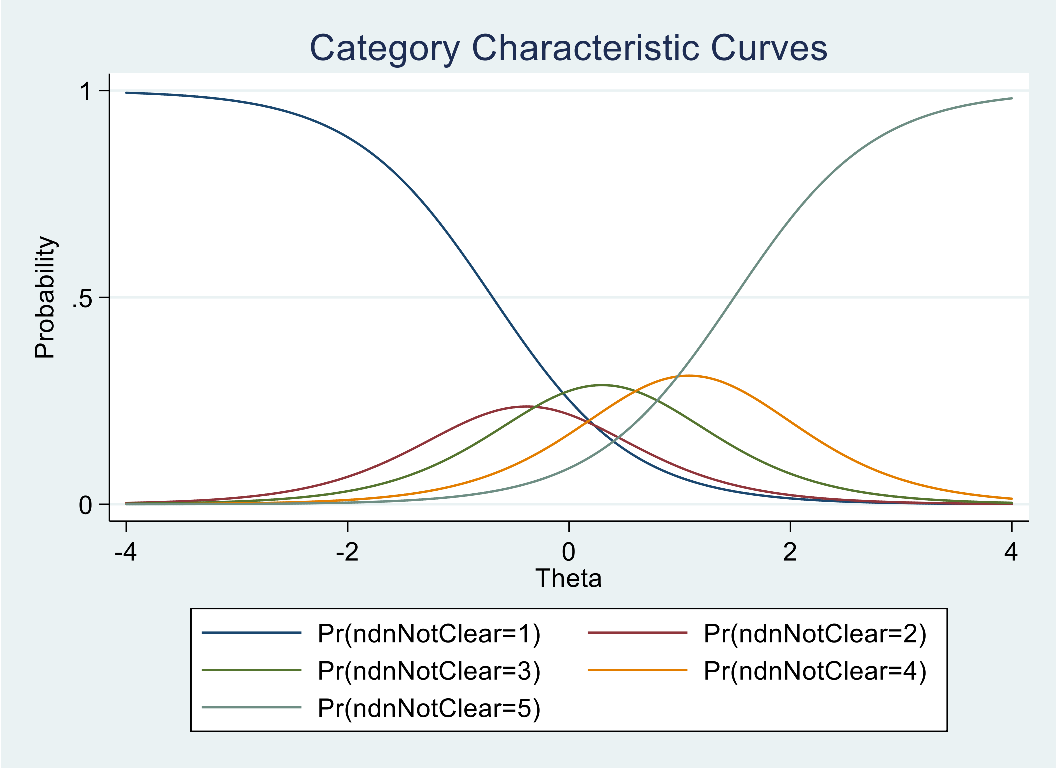

Item 6. I didn't know what the symptom meant

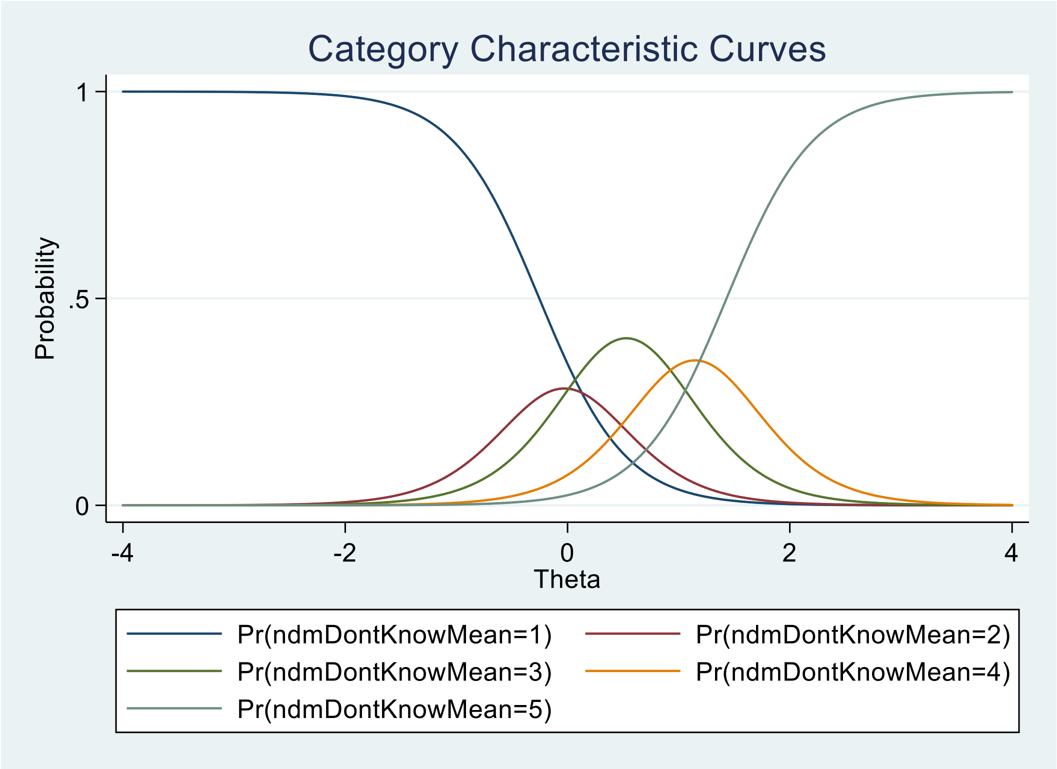

Item 9. I thought the symptom might be due to something else

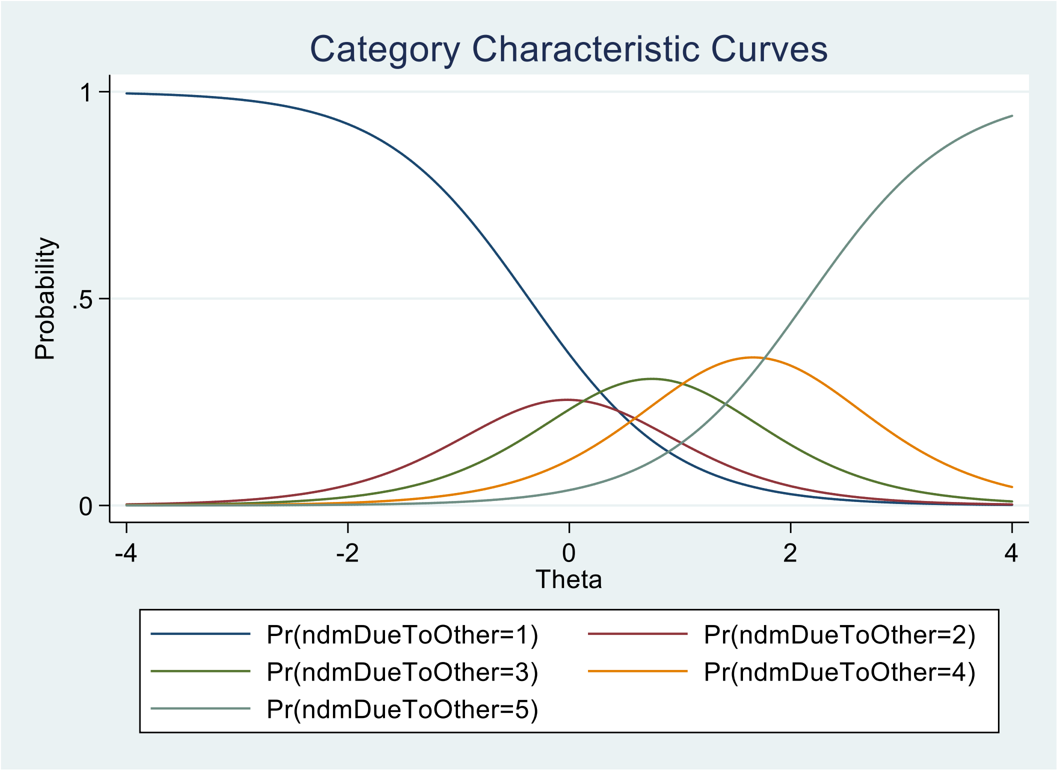

Item 14. I wasn't sure how important the symptom was

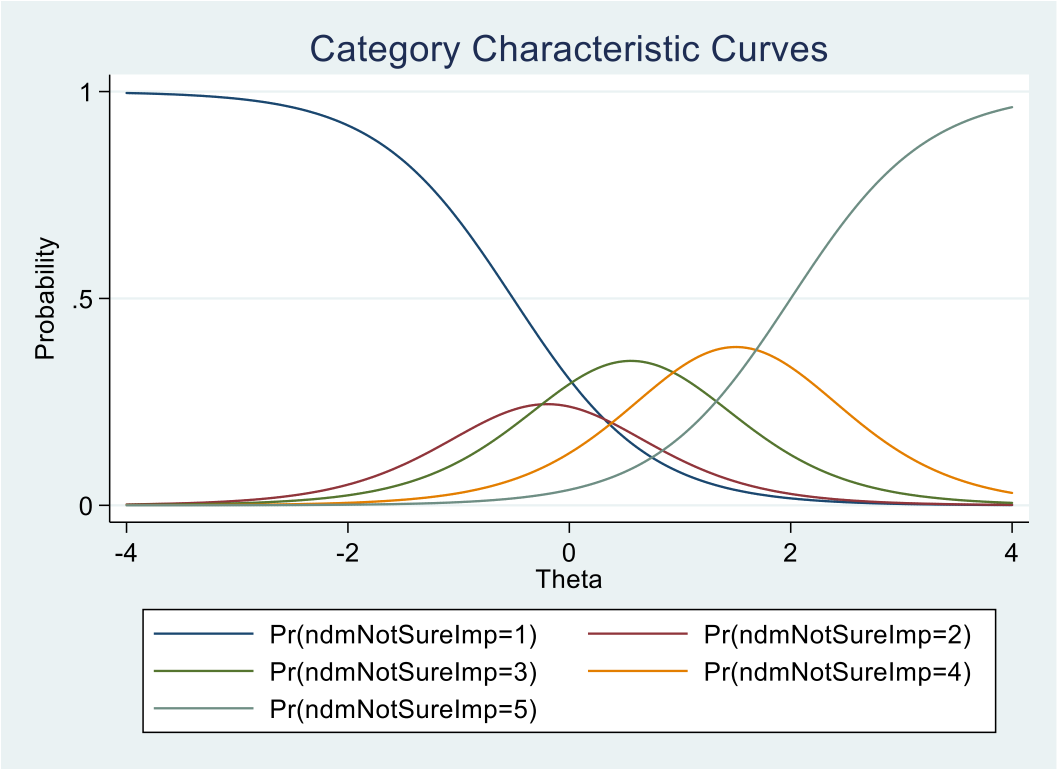

Item 17. When I had the symptom, I didn't understand what was happening

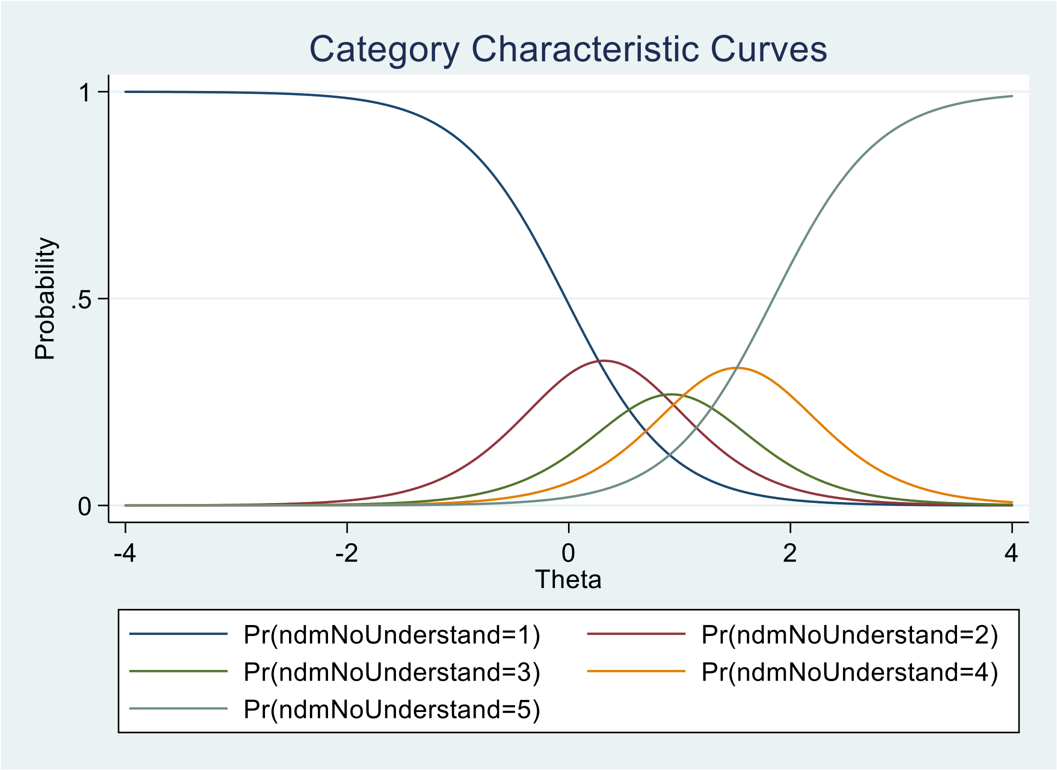

Item 22. The symptom was new to me

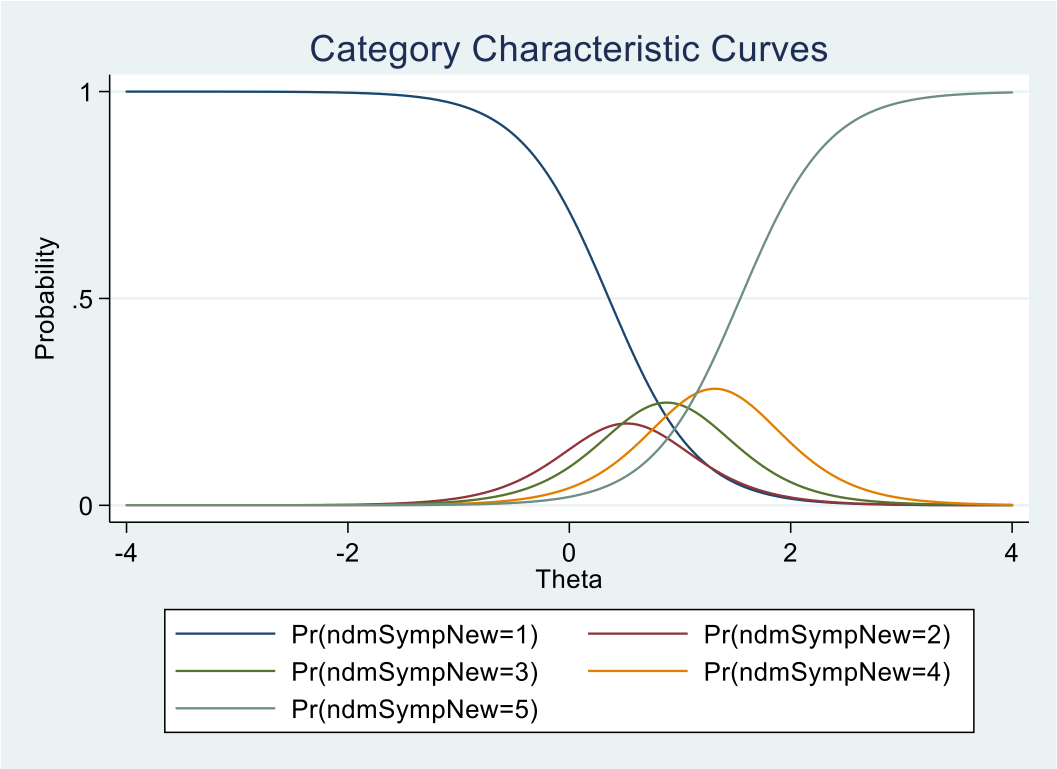

Item 33. I recognized this symptom from the last time I had it

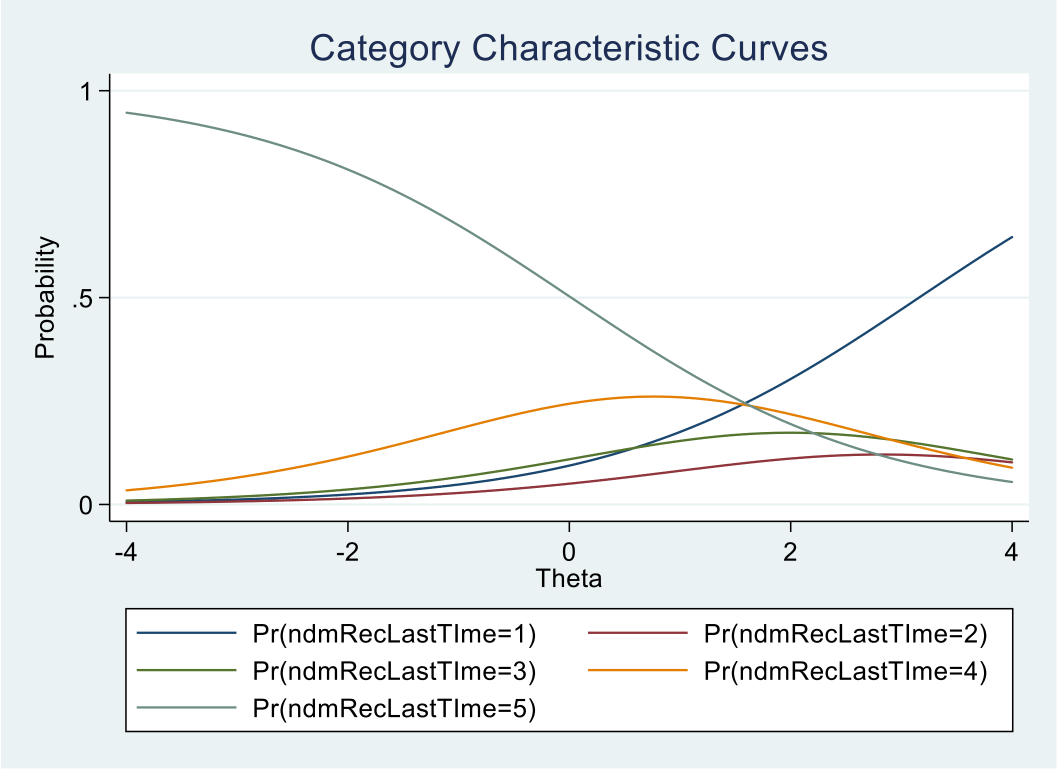

Item 35. The symptom was different than the last time I had it

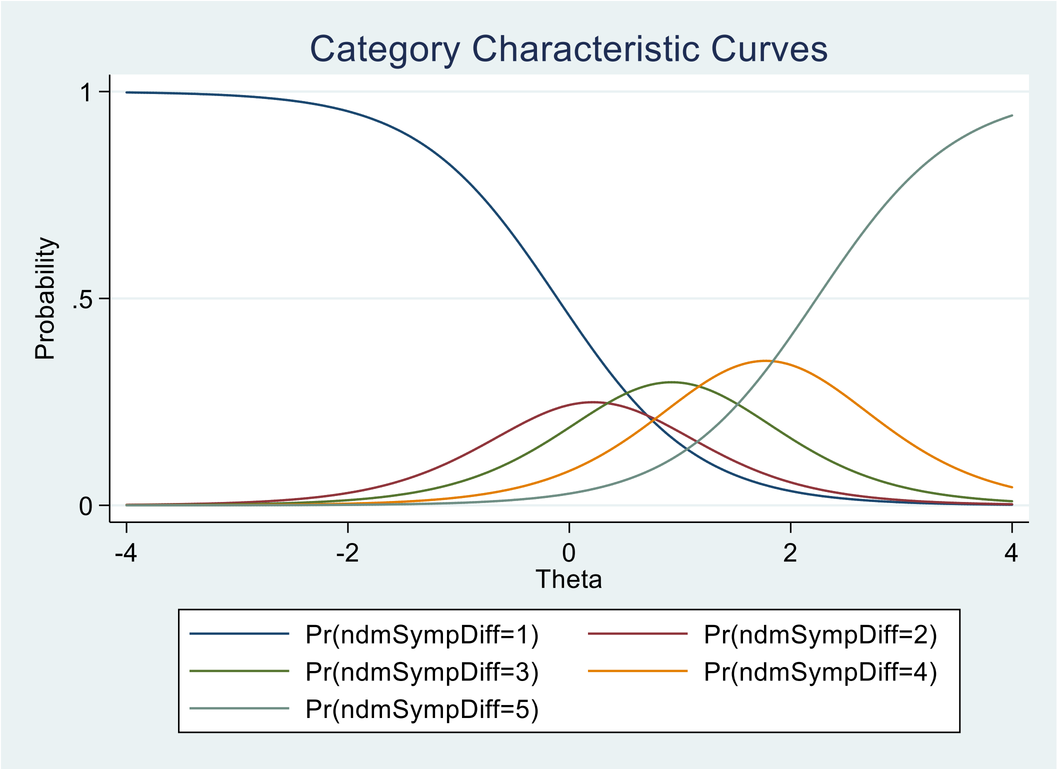

Cognitive/Affective Scale

Item 8. I felt too sad to make a decision

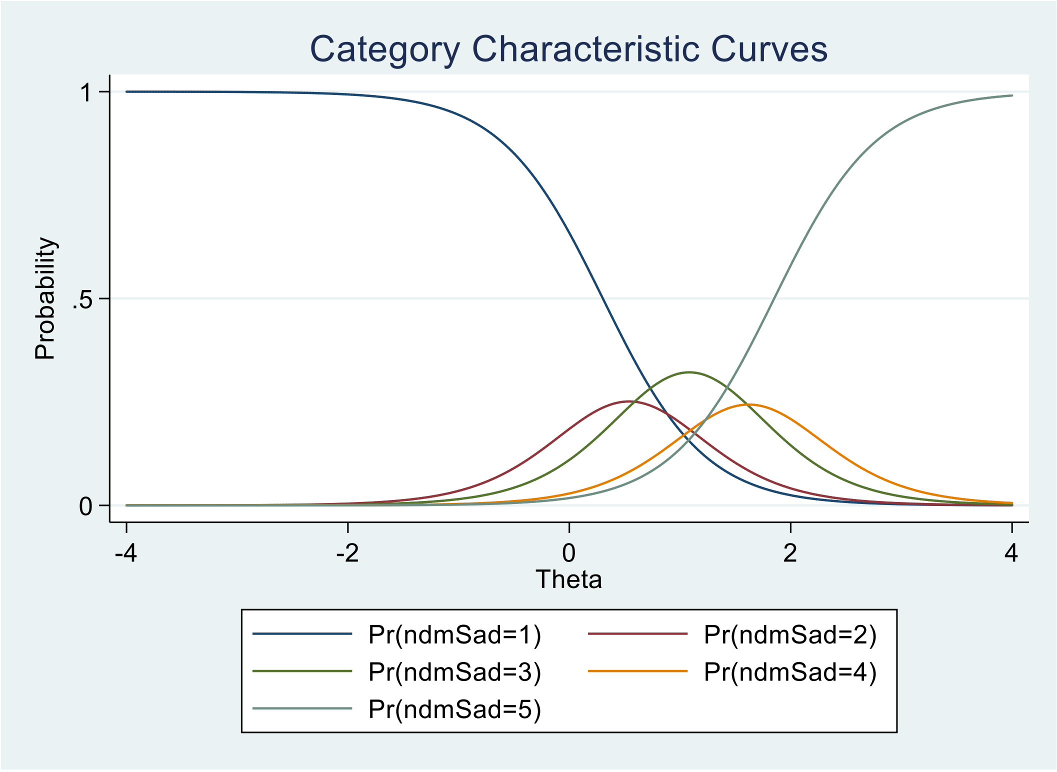

Item 15. My thinking was not clear so I could not make a decision

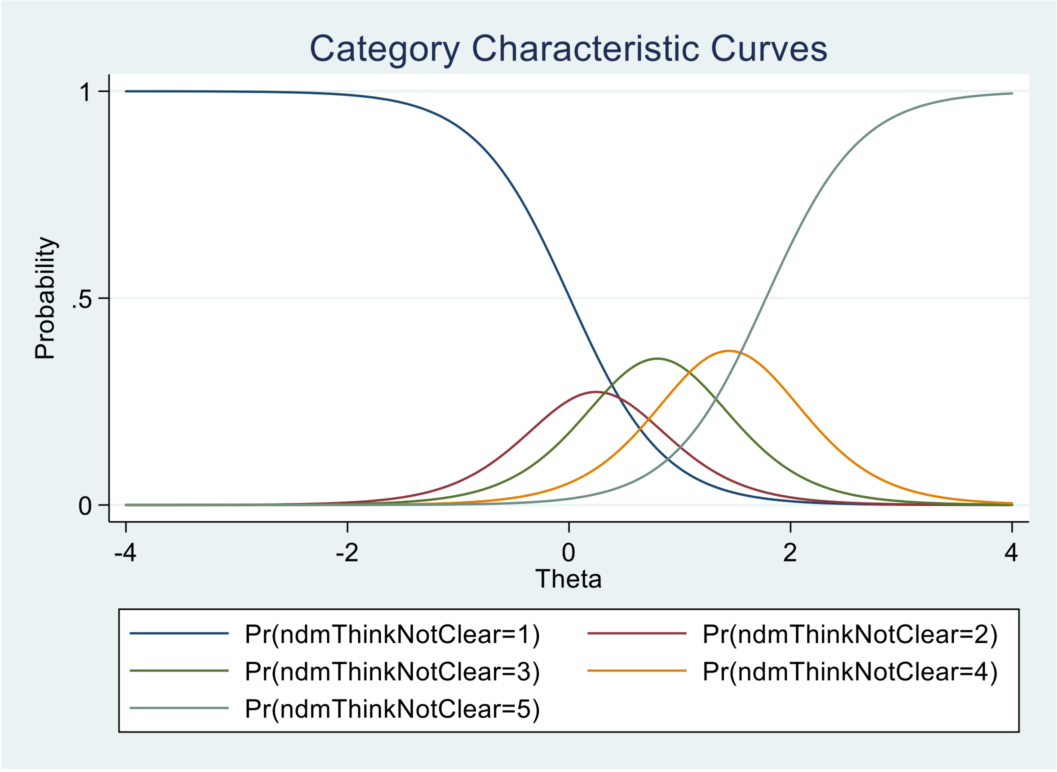

Item 21. I felt too anxious to make a decision

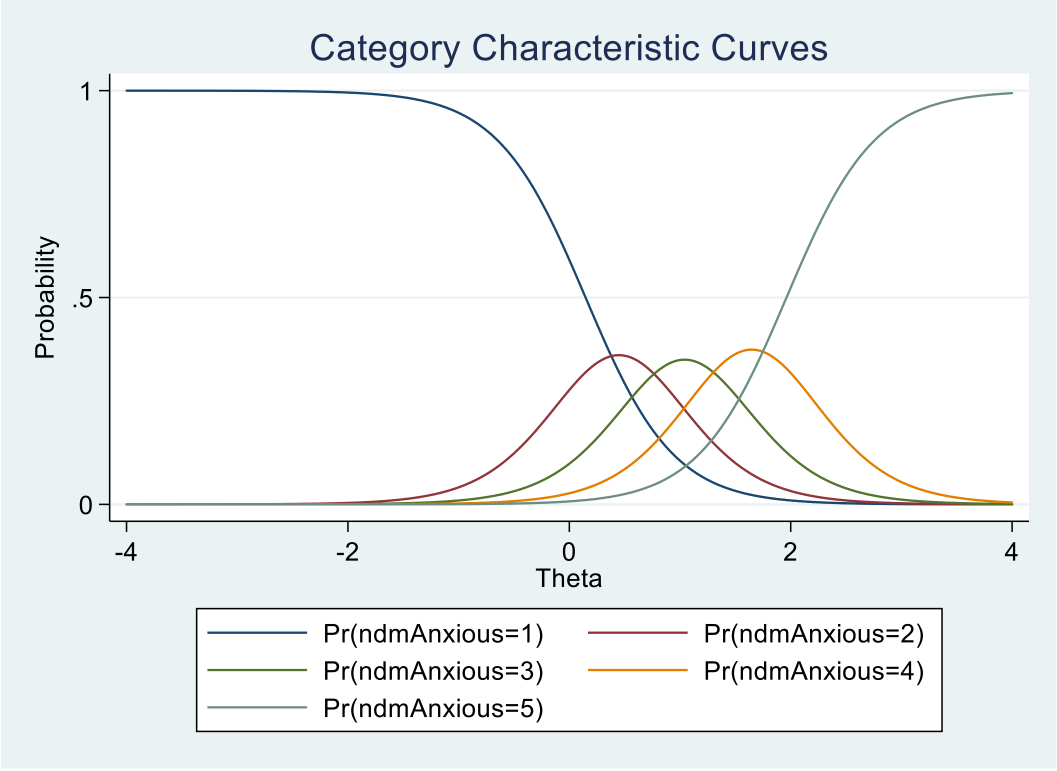

Item 26. I didn't feel well enough to make a decision

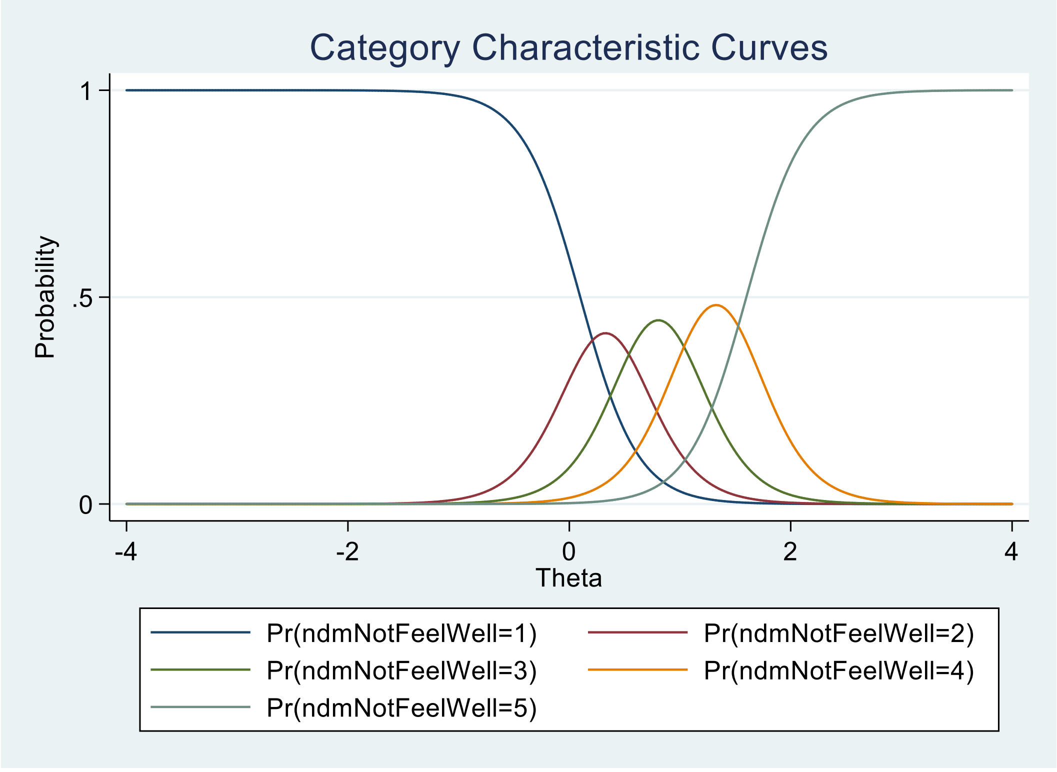

Item 31. I felt too tired to make a decision

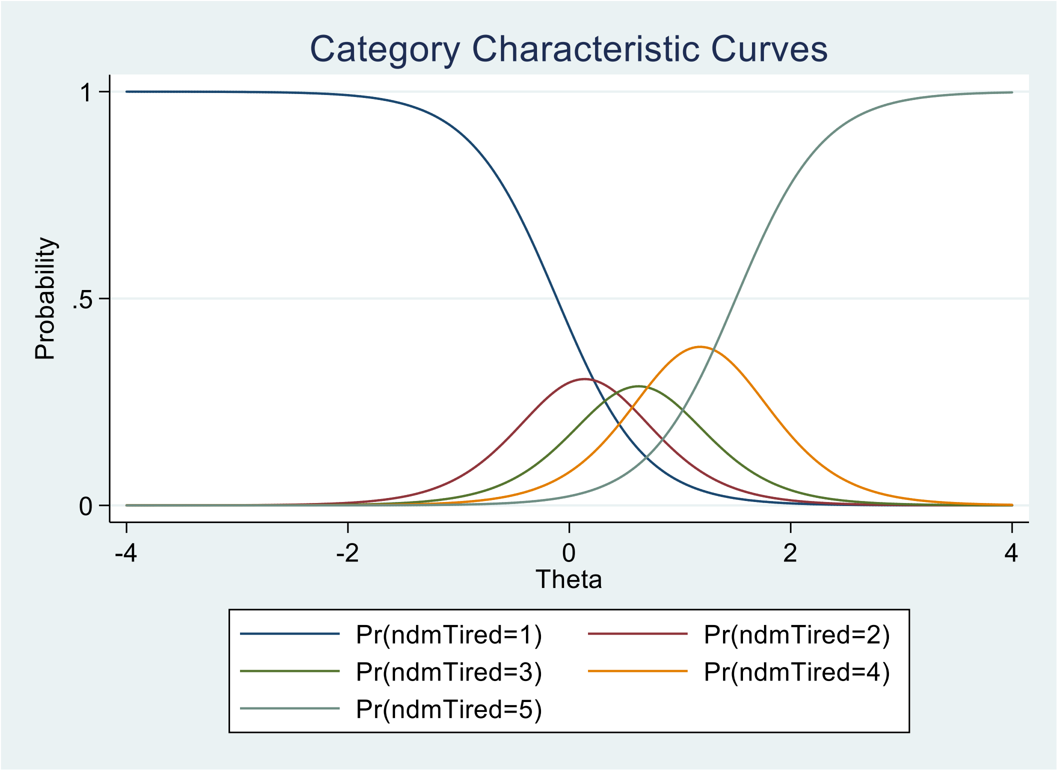

Item 32. I felt uncertain about what to do

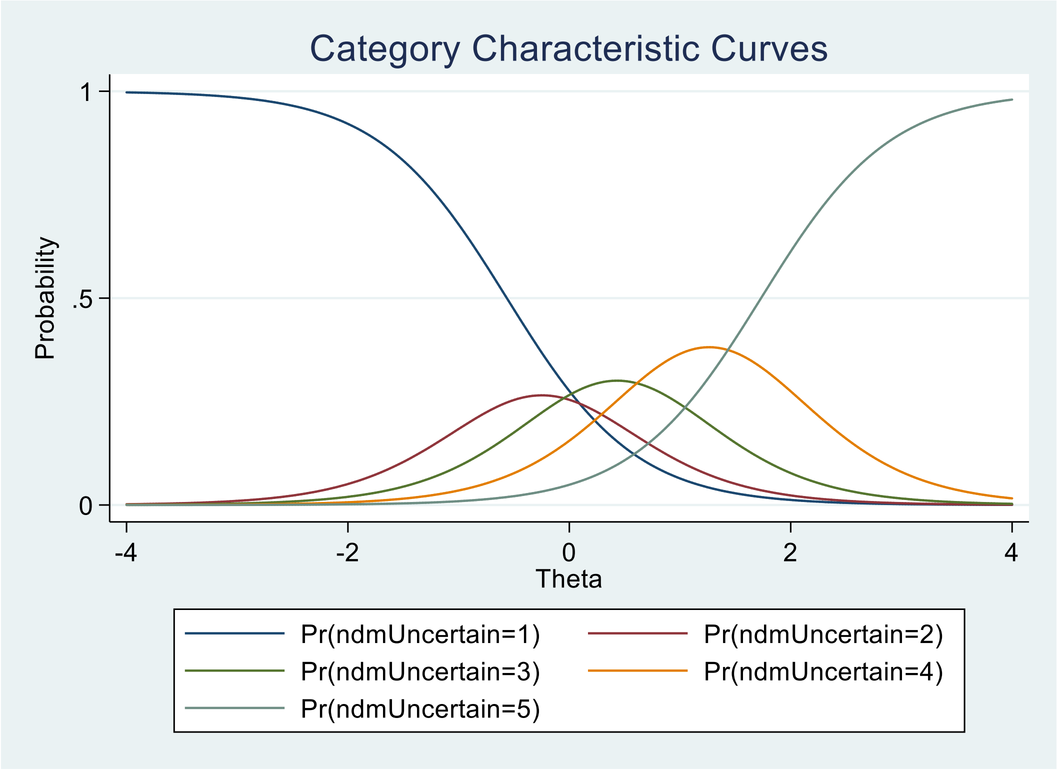

Waiting/Cue Competition Scale

Item 16. I thought I could wait to make a decision

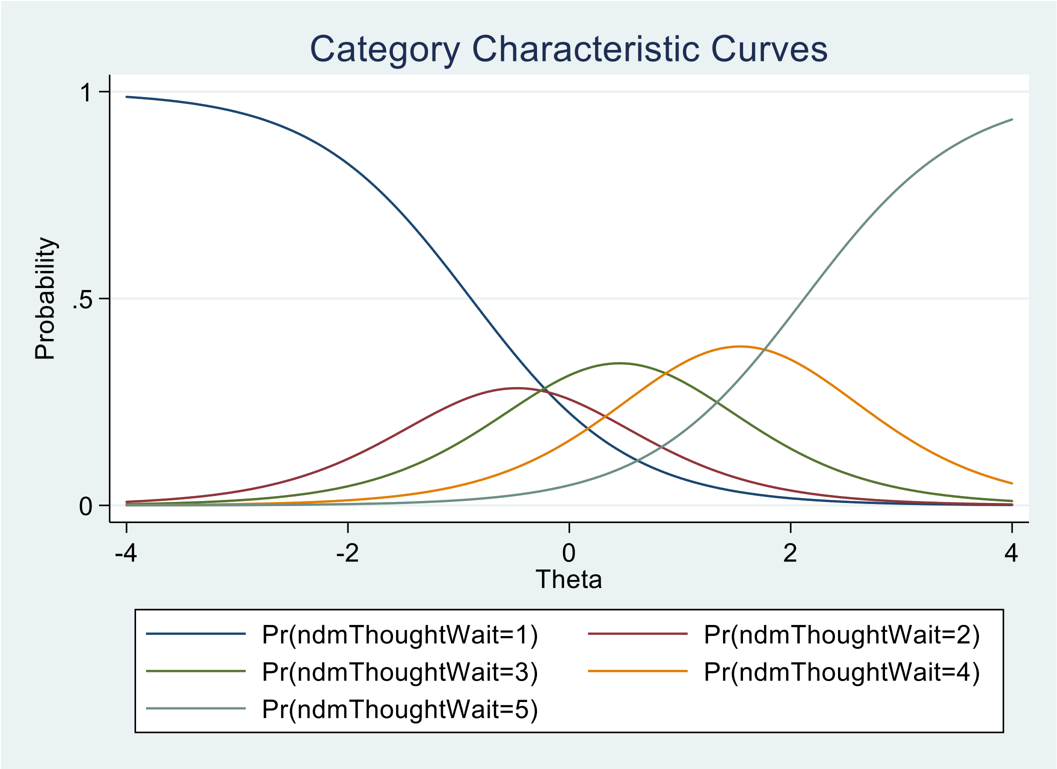

Item 24. I felt that the symptom was nothing to worry about

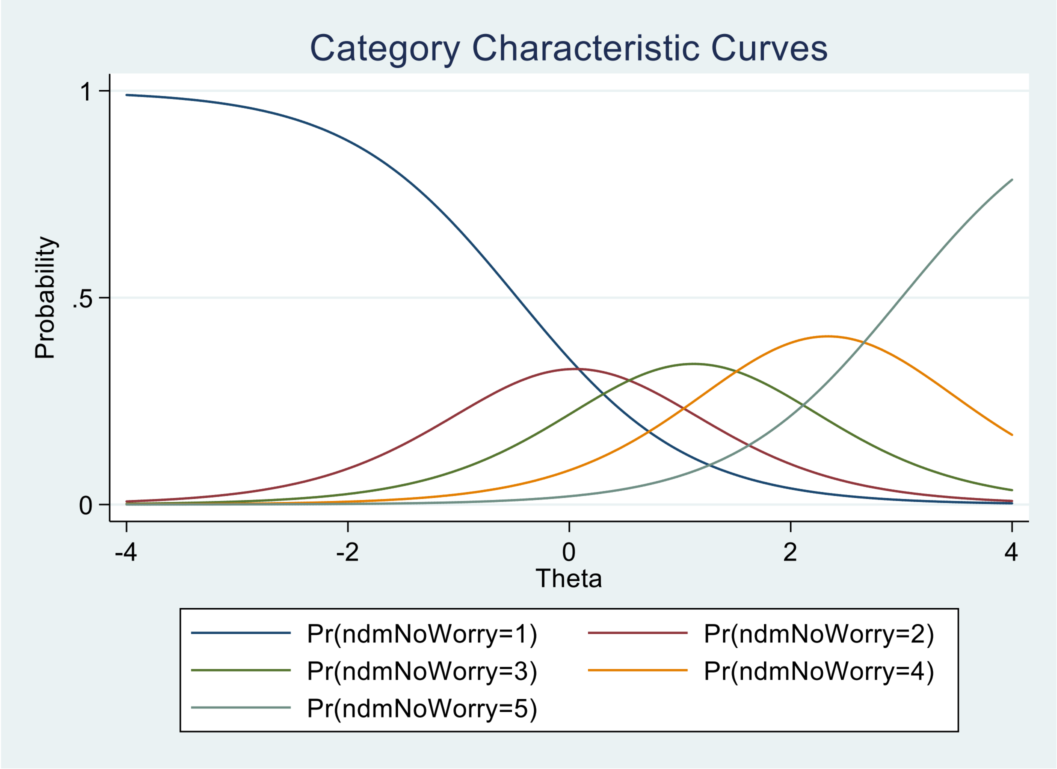

Item 25. The symptom changed slowly

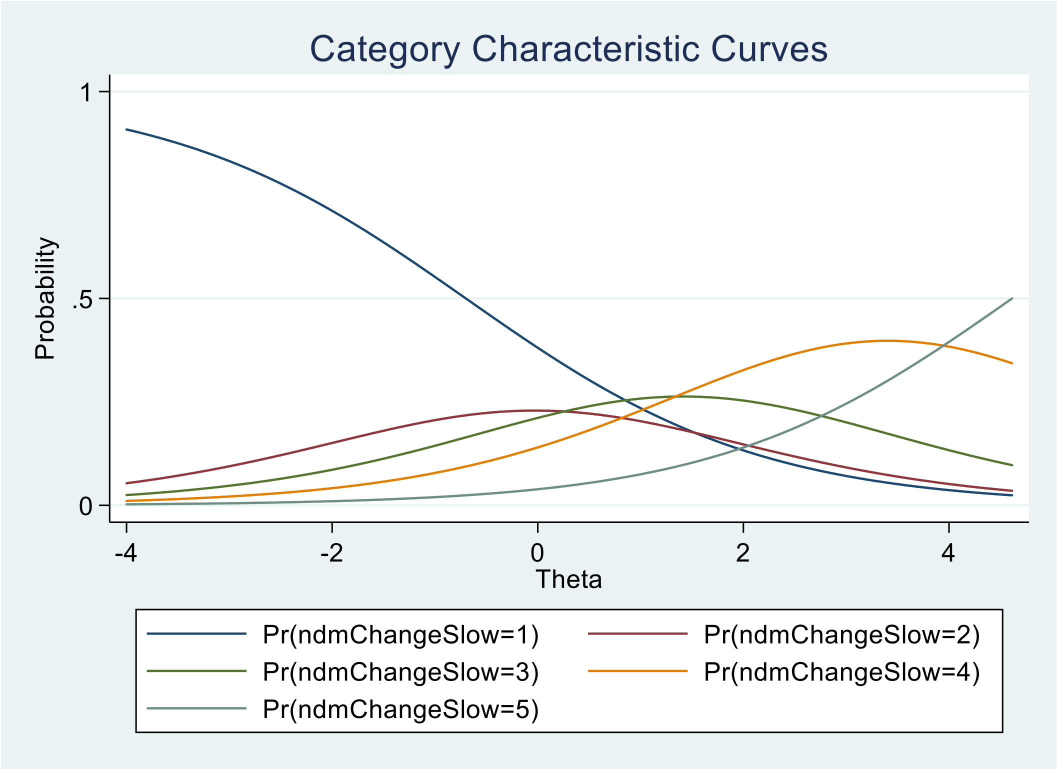

Item 27. I thought I could tolerate the symptom

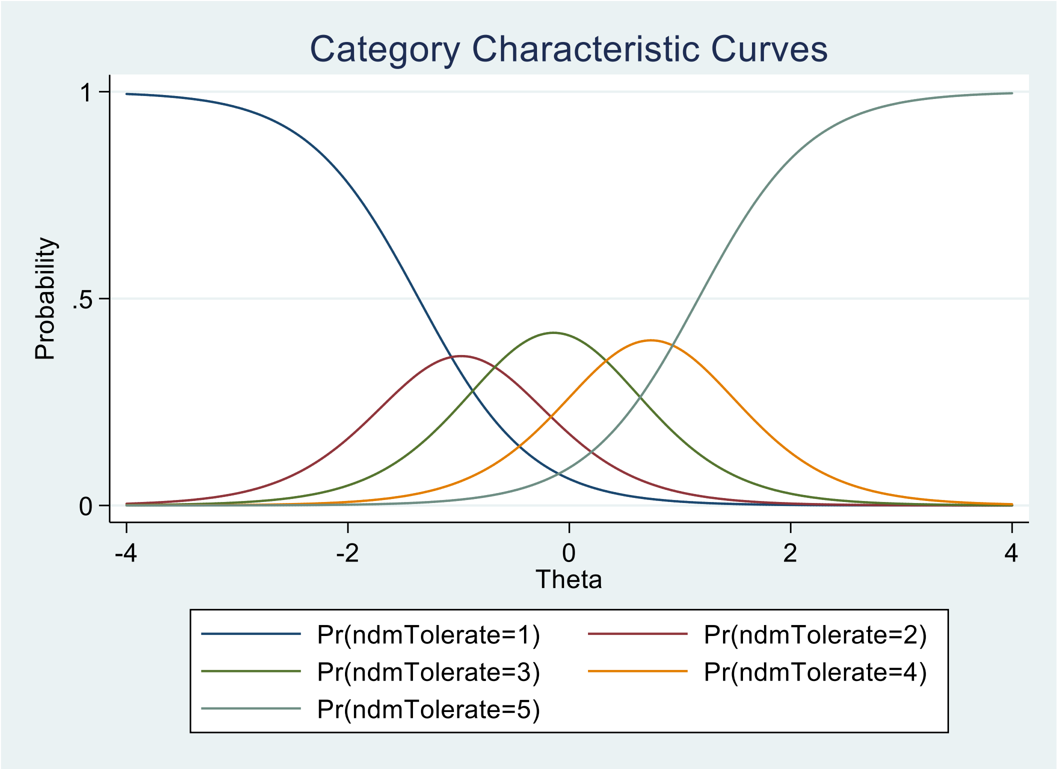

Item 29. Someone else needed my attention

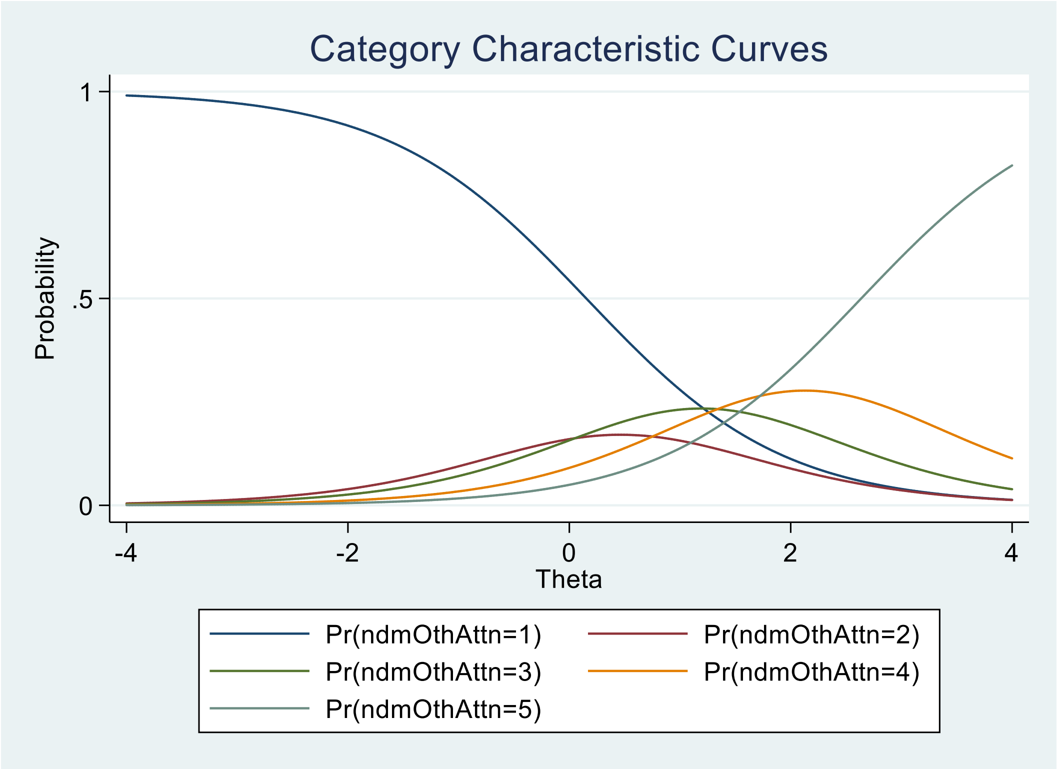

Item 30. I thought the symptom would go away on its own

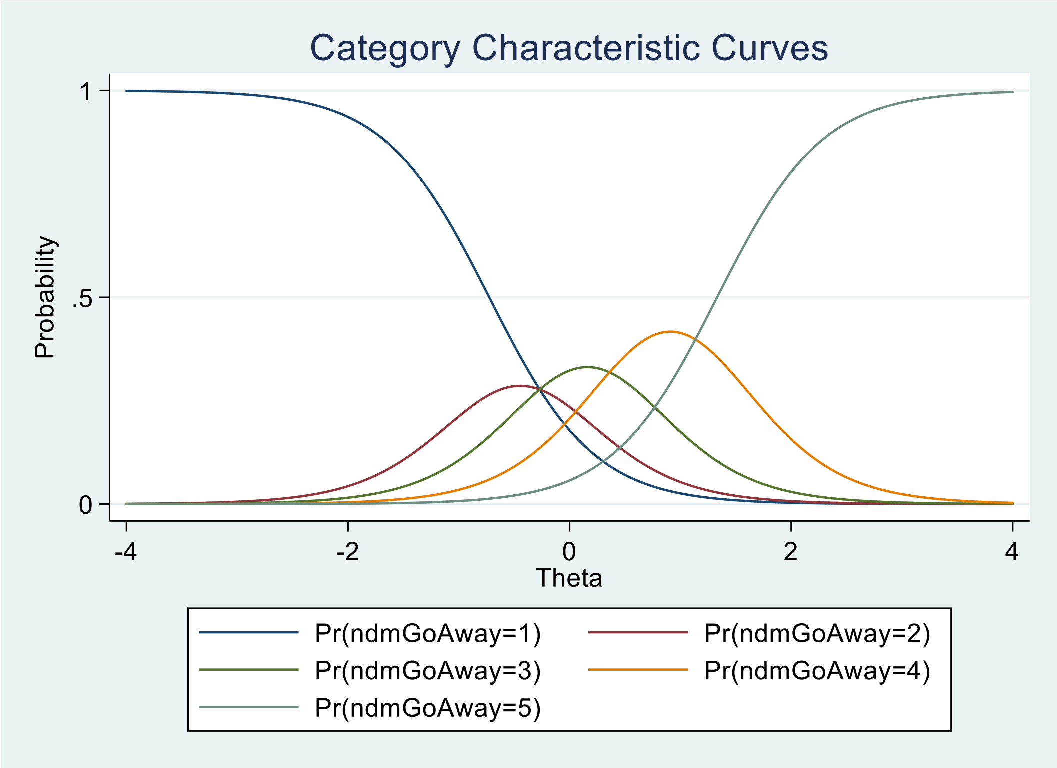

Item 34. Other things were more important at the time

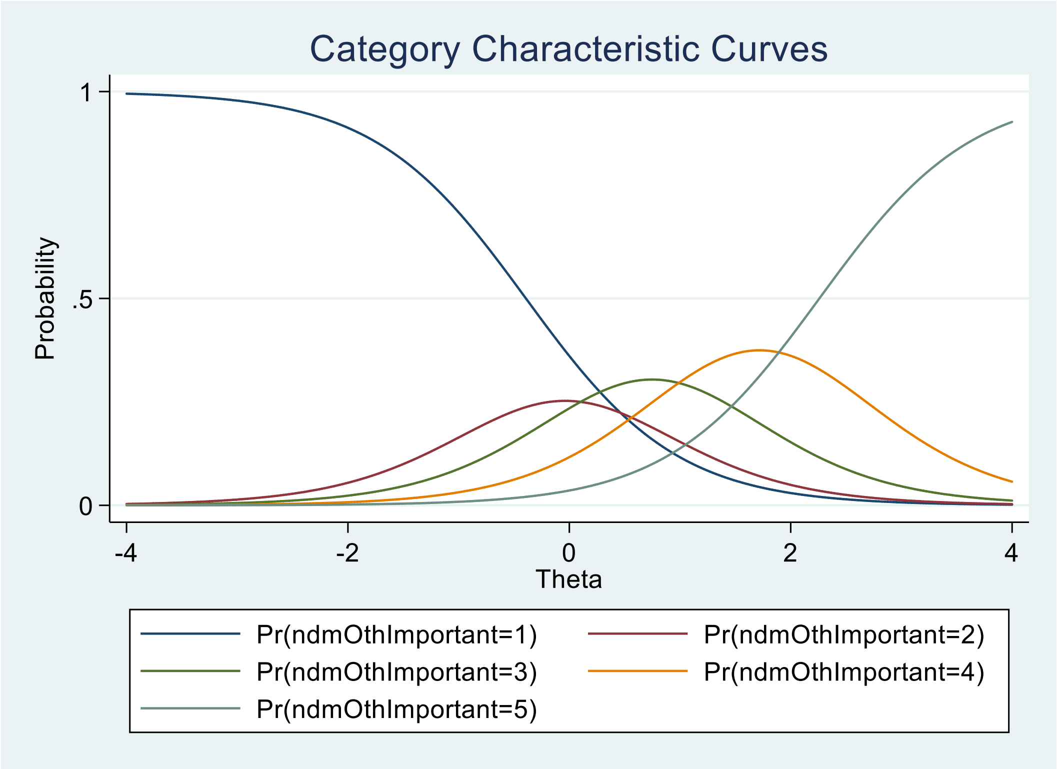

Concealment Scale

Item 11. I felt embarrassed about my symptom

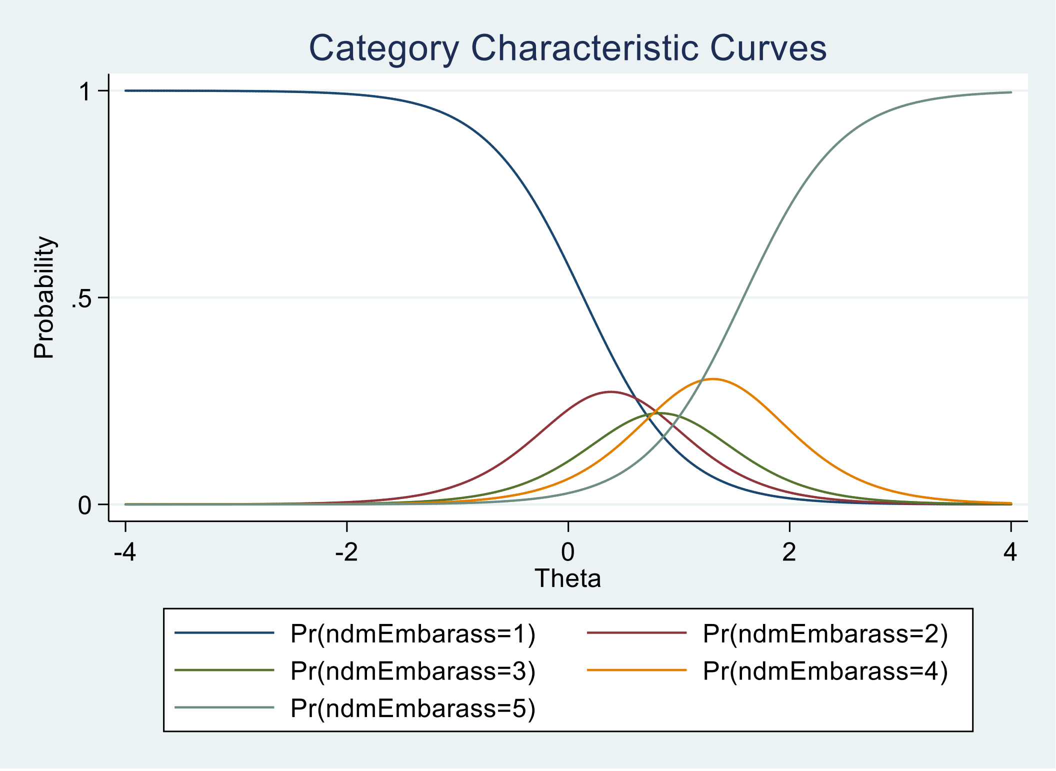

Item 12. I didn't want to burden my family

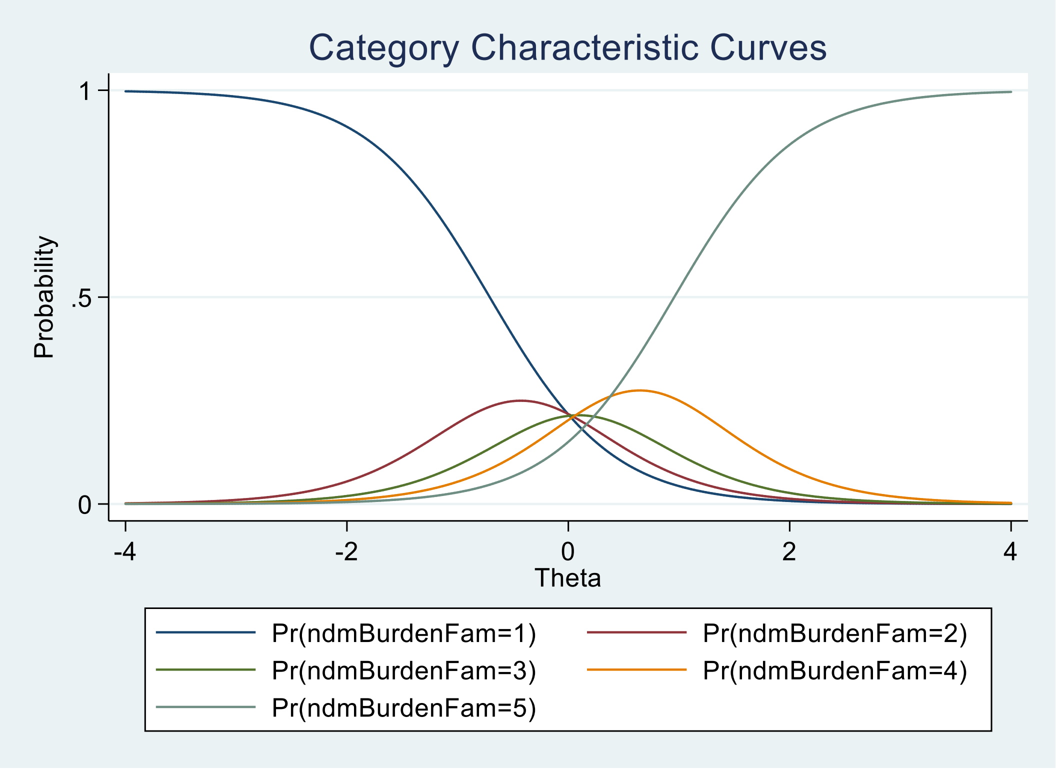

Item 23. I didn't want people to know about my symptom

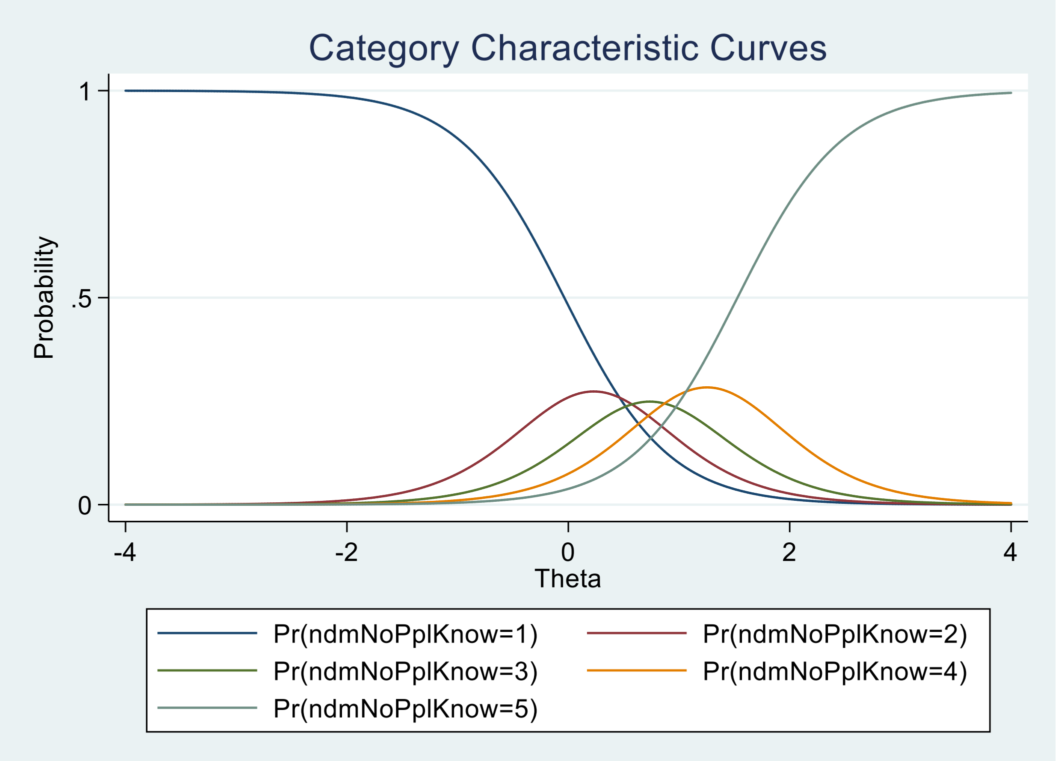

Supplement: Supplementary file 2 — Additional file 2: Category characteristic curves for each item. [file 12955_2022_1990_MOESM2_ESM.pdf]
